# Conformation-dependent lesion bypass of bulky arylamine-dG adducts generated from 2-nitrofluorene in epigenetic sequence contexts

**DOI:** 10.1093/nar/gkad1038

**Published:** 2023-11-11

**Authors:** Alicia M Crisalli, Yi-Tzai Chen, Ang Cai, Deyu Li, Bongsup P Cho

**Affiliations:** Department of Biomedical and Pharmaceutical Sciences, College of Pharmacy, University of Rhode Island, Kingston, RI 02881, USA; Department of Biomedical and Pharmaceutical Sciences, College of Pharmacy, University of Rhode Island, Kingston, RI 02881, USA; Department of Biomedical and Pharmaceutical Sciences, College of Pharmacy, University of Rhode Island, Kingston, RI 02881, USA; Department of Biomedical and Pharmaceutical Sciences, College of Pharmacy, University of Rhode Island, Kingston, RI 02881, USA; Department of Biomedical and Pharmaceutical Sciences, College of Pharmacy, University of Rhode Island, Kingston, RI 02881, USA

## Abstract

Sequence context influences structural characteristics and repair of DNA adducts, but there is limited information on how epigenetic modulation affects conformational heterogeneity and bypass of DNA lesions. Lesions derived from the environmental pollutant 2-nitrofluorene have been extensively studied as chemical carcinogenesis models; they adopt a sequence-dependent mix of two significant conformers: major groove binding (B) and base-displaced stacked (S). We report a conformation-dependent bypass of the *N*-(2′-deoxyguanosin-8-yl)-7-fluoro-2-aminofluorene (dG-FAF) lesion in epigenetic sequence contexts (d[5′-CTTCTC^#^G*NCCTCATTC-3′], where C^#^ is C or 5-methylcytosine (5mC), G* is G or G-FAF, and N is A, T, C or G). FAF-modified sequences with a 3′ flanking pyrimidine were better bypassed when the 5′ base was 5mC, whereas sequences with a 3′ purine exhibited the opposite effect. The conformational basis behind these variations differed; for -CG*C- and -CG*T-, bypass appeared to be inversely correlated with population of the duplex-destabilizing S conformer. On the other hand, the connection between conformation and a decrease in bypass for flanking purines in the 5mC sequences relative to C was more complex. It could be related to the emergence of a disruptive non-S/B conformation. The present work provides novel conformational insight into how 5mC influences the bypass efficiency of bulky DNA damage.

## Graphical Abstract

**Graphical Abstract:**
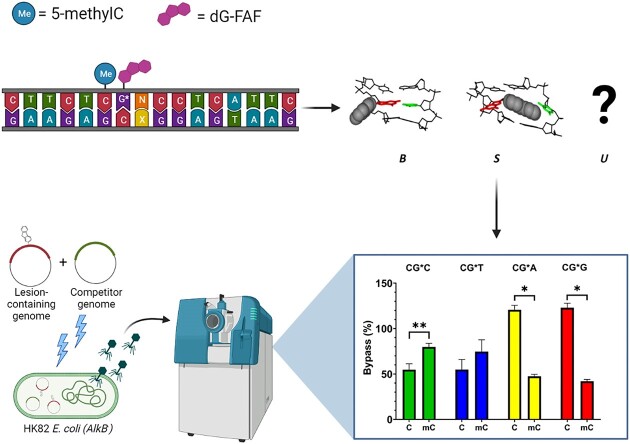

## Introduction

**Figure 1.**
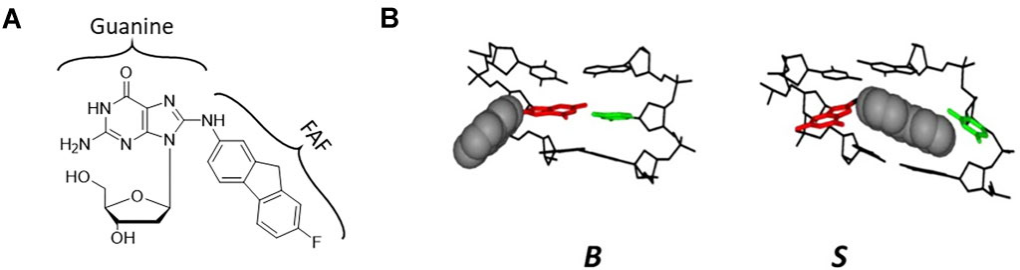
(**A**) Structure of the *N*-(2′-deoxyguanosin-8-yl)-7-fluoro-2-aminofluorene (dG-FAF) adduct and (**B**) molecular models of the major conformations of FAF-adducted DNA: major groove binding (B) and base-displaced stacked (S). Guanine (center base on left strand) is highlighted in red sticks, the opposite C (center base on right strand) is highlighted in green sticks and the bulky FAF adduct is shown in gray (spacefill model). Adapted with permission from ref. (8). Copyright 2012 American Chemical Society.

The sequence context of a DNA lesion strongly influences its structural characteristics, particularly conformational heterogeneity, and subsequent toxicity (1–7). There are three predominant conformations a bulky arylamine lesion can adopt, namely major groove binding (B-type), base-displaced stacked (S-type) and minor groove wedge (W-type) (8). DNA adducts generated from the environmental pollutant 2-nitrofluorene (Figure 1A) are extensively studied as models for the chemical carcinogenesis of arylamines and nitroarenes, adopting a sequence-dependent mixture of B and S conformations (Figure 1B) (9–14). The highly mutagenic dG-AAF [*N*-(2′-deoxyguanosin-8-yl)-2-acetylaminofluorene] lesion in the *NarI* recognition sequence (5′-G_1_G_2_CG_3_*CC-3′), a mutational hotspot, mainly induces −2 frameshift mutations. This is caused by deletion of the modified CpG* dinucleotide in translesion synthesis, dependent on the polymerase involved (Pol II versus Pol V) (15,16). Although there are three possible dG adduct sites, the −2 deletion only occurs when the adduct is on G_3_, the CpG site, likely due to a localized conformational anomaly (17). This was confirmed by nuclear magnetic resonance (NMR) and modeling data showing that the larger bulk of dG-AAF relative to dG-AF [*N*-(2′-deoxyguanosin-8-yl)-2-aminofluorene] promotes the *syn* orientation of the adducted guanine (stacked conformation), stabilizing a slipped replication intermediate, which promotes the −2 deletion (18). While dG-AF can also induce similar frameshift mutations, it more frequently causes point mutations due to its less bulky structure (19,20). It is unknown, however, how a methylated CpG (meCpG) in this sequence would influence lesion bypass and mutagenesis.

While 5-methylcytosine (5mC) is a common epigenetic DNA modification (0.75–1.00% of mammalian DNA depending on tissue type) (21,22) and dysregulation of DNA methylation is a known biomarker for many cancers (23), it is not well understood, especially *in cell*, how 5mC affects structure/conformation and subsequent mutagenicity of a bulky DNA adduct as a flanking base. One study by Watt *et al.* found that the frequency of mutations doubled when 5mC was 5′ to the *N*-(deoxyguanosine-8-yl)-1-aminopyrene lesion on the second base of codon 273 in the p53 tumor suppressor gene in mammalian cells compared to C. They also found that the major mutations varied depending on cell type (24). Moreover, Feng *et al.* found that meCpG sites in the human p53 gene not only augmented the formation of 4-aminobiphenyl (ABP)-derived lesions but also created binding sites out of CpGs that were previously not favored (25). The structural basis for this increase in lesion formation and mutagenicity is unknown, however, as there were no accompanying, robust structure–activity studies. Other studies have found that the reactivity of guanine to form (+)-*anti*-benzo[*a*]pyrene 7,8-dihydrodiol 9,10-epoxide (anti-BPDE) adducts increased in some sequence contexts when C was replaced with 5mC. It was also determined by ultraviolet (UV) melting that modified duplexes containing 5mC were more thermally stable, and conformational heterogeneity was suggested via circular dichroism (CD) spectroscopy and UV absorption, but no accompanying high-resolution conformational studies (i.e. NMR) were conducted (26,27). One NMR conformational study found that 5mC affects the two enantiomers of *anti*-BPDE differently; the 10*R*-(−)-*trans*-*anti*-[BP]G adduct shifts from a minor groove W-type conformation in the unmethylated CpG context to a base-displaced intercalative S-type conformation in the meCpG context, whereas the 10*S*-(+)-*trans*-*anti*-[BP]G remained in the minor groove conformation regardless of CpG methylation (28). How such significant conformational transitions can affect the processing of the lesions by repair and replicative mechanisms inside the cell is unknown.

The present study aimed to parse the lesion-induced conformation–activity relationship behind the mutagenicity and replication bypass effects of bulky DNA adducts in epigenetically relevant (5mC/meCpG-containing) sequence contexts using the isosteric fluorine-containing model lesion FAF (Figure 1A). Herein, we site-specifically modified eight sequences of 16mer oligonucleotides (d[5′-CTTCTC^#^G*NCCTCATTC-3′], where C^#^ is C (control) or 5mC, G* is dG (control) or dG-FAF, and *N* is A, T, C or G) and systematically varied the 3′ flanking base to the lesion site to create a complete set of oligonucleotides in which the lesion was always preceded by either C or 5mC and followed by all of the four standard bases, A, T, C or G, in the 5′ → 3′ direction (henceforth denoted by their core sequence -C^#^G*N-). These template strands were then annealed to complementary strands for analysis of the duplex structure and B/S conformational heterogeneity by 1D, 2D and ^19^F NMR, CD spectroscopy, thermal stability by optical melting experiments and in-cell lesion bypass (competitive replication of adduct bypass, CRAB) and mutagenicity (restriction endonuclease and post-labeling, REAP) assays (29–31).

## Materials and methods

*Warning*: 2-Nitrofluorene and its derivatives are known mutagens and animal carcinogens, and therefore should be handled with caution.

### Preparation of site-specifically modified oligonucleotides

Desalted oligonucleotides (oligos, 2–5 μmol scale) and primers were purchased from Integrated DNA Technologies (Coralville, IA). Oligos with the sequence d[5′-CTTCTC^#^G*NCCTCATTC-3′] (where C^#^ is C or 5mC, G* indicates the lesion position, and N is A, T, C or G) were site-specifically modified following established procedures (details are provided in the Supplementary Methods, Supplementary Figures S1–S3 and Supplementary Table S1) (2,10,32,33). Ultimately, 16 sequences were synthesized and purified for analysis: 8 unmodified controls and 8 modified with dG-FAF.

### NMR spectroscopy

^19^F NMR samples were prepared by annealing the FAF-modified 16mer oligos (100 μM) with an equimolar amount of the corresponding 16mer complementary strands in 100% D_2_O at 85°C for 5 min and then slowly cooling to room temperature over 3–4 h. ^19^F NMR [1D, chemical exchange saturation transfer (CEST), exchange spectroscopy (EXSY)] was recorded at 25°C on a Bruker Avance III HD 600 MHz Spectrometer equipped with a 5 mm cryoprobe QXI (^1^H{^19^F/^13^C/^15^N}) *Z*-axis operating at 564.4 MHz. B/S conformational ratios were quantified by integration of the fitted 1D spectra. Imino ^1^H NMR samples were prepared and annealed in an identical manner to the ^19^F NMR samples in 10% D_2_O:90% H_2_O. Imino proton spectra were recorded at 3°C on a Bruker Avance III HD 600 MHz Spectrometer equipped with a 5 mm cryoprobe QXI (^1^H{^19^F/^13^C/^15^N}) *Z*-axis operating at 600 MHz. FIDs (free induction decay) were processed using MestReNova software version 14.1.0-24037 (Mestrelab Research S.L., Santiago, Spain) with exponential line broadening and Fourier transformation. More details can be found in the Supplemental Methods.

### CD spectroscopy

Modified and unmodified 16mer templates (20 μM) were annealed to an equimolar amount of primers of varying length (8mer–12mer and 16mers) spanning *n* − 2 to *n* + 2 (where *n* is the lesion position) and the fully paired duplexes, in 800 μl CD/UV buffer (0.2 M NaCl, 10 mM sodium phosphate, 0.2 mM EDTA, pH 7.0) following the procedure described above. CD measurements were recorded on a Jasco J-1100 CD Spectrometer (JASCO, Inc., Easton, MD) equipped with temperature control scanning from 400 to 200 nm at 50 nm/min for five cycles at 25°C. Raw data were baseline subtracted and smoothed using adaptive algorithms provided by Jasco Spectra Manager Version 2 Spectra Analysis software (version 2.14.00).

### UV melting experiments

UV melting experiments were carried out on a Cary 100 Bio UV-visible spectrophotometer equipped with temperature control, a 12-cell sample changer and 1 cm path length, self-masking quartz cuvettes. Control and modified duplexes (0.8–4.0 μM) were prepared in CD/UV buffer (0.2 M NaCl, 10 mM sodium phosphate, 0.2 mM EDTA, pH 7.0) and heated from 15 to 85°C at a ramp rate of 1°C/min monitoring at 260 nm for five cycles. Melting temperatures and thermodynamic parameters (Δ*H*, Δ*S*, Δ*G*) were calculated from the melting curves using Meltwin 3.5 software.

### Lesion bypass and mutagenesis in cell

#### M13 genome construction

Oligonucleotides (16mer) containing lesions and unmodified controls (Supplementary Tables S3 and S4) were phosphorylated, annealed with scaffolds and ligated into 58mer or 61mer oligonucleotides, which were further ligated into M13mp7(L2) single-stranded viral genome. The constructed genomes were purified and recovered. The scheme of general M13 construction procedure is shown in Supplementary Figures S23 and S29 (29,31).

#### In-cell bypass and mutagenesis assays

The relative bypass (replication efficiency) of the FAF lesion was measured in HK82 (AlkB^−^) *Escherichia coli* using the CRAB assay; mutational analysis was performed by using the REAP assay as previously described (29,31,34,35). Lesion bypass, the ratio between the intensities of the modified and competitor fragments, was determined and normalized to the ratio obtained from the similar experiment employing a lesion-free ‘G’ control, considered as 100% bypass. All data represent the mean ± standard deviation of three independent experiments. The 1,*N*^6^-ethenoadenine (eA) containing M13 was used as a control to validate the methods (Supplementary Figure S31) (30). One-way analysis of variance and least significant difference post hoc multiple comparison test were used to explore sources of variance. An unpaired Student’s *t*-test was used to assess statistical significance; *P* < 0.05 was considered significant. Detailed assay parameters can be found in the Supplemental Methods; mass spectrometry, gel electrophoresis and polymerase chain reaction data for the CRAB and REAP are shown in Supplementary Figures S22, S24–S28 and S30 and Supplementary Table S5, and an overview diagram of the CRAB and REAP assays is shown in Supplementary Figure S29.

## Results

### dG-FAF as a conformational probe

2-Nitrofluorene is a nitroarene pollutant found in diesel exhaust, listed as a Group 2B human carcinogen by the World Health Organization’s International Agency for Research on Cancer (36). It is metabolized to reactive hydroxylamine intermediates by nitroreductase and excreted in urine as glucuronide or sulfate conjugates. The latter electrophile produces DNA adducts dG-AF and dG-AAF. Both adducts have been studied extensively as prototype bulky arylamine–DNA adducts (37).

We used dG-FAF as a probe for conformational assessment in the present study (Figure 1). Incorporation of fluorine at the 7-position of AF has been shown to maintain comparable carcinogenicity (38,39) and adduct conformational profiles to the nonfluorinated species (40–42). Meneni *et al.* has shown *E. coli* UvrABC nucleotide excision repair (NER) incision efficiency of dG-AF versus dG-FAF on the 12mer sequence 5′-CTTCTAGG*CCTC-3′, to be essentially identical, indicating their comparable conformational characteristics and NER responses (32,43). These results ensured that the conformational and biological results observed with FAF could be extrapolated to those of nonfluorinated dG-AF adducts.

Due to our biomimetic approach for modifying the oligonucleotides, the template sequence used herein necessarily contains only one guanine residue. It is consequently pyrimidine-rich, as adenine is also susceptible to modification by the electrophilic lesion precursor. Incorporating mostly cytosine keeps the C/G content, and therefore the stability, of the duplex high, with the complementary strand being G-rich. As the S/B conformations only exist in duplex and purine-to-pyrimidine content is balanced in this context, the enrichment of pyrimidine in the lesion-containing sequence should not be a significant problem (44).

### Conformational heterogeneity

**Figure 2.**
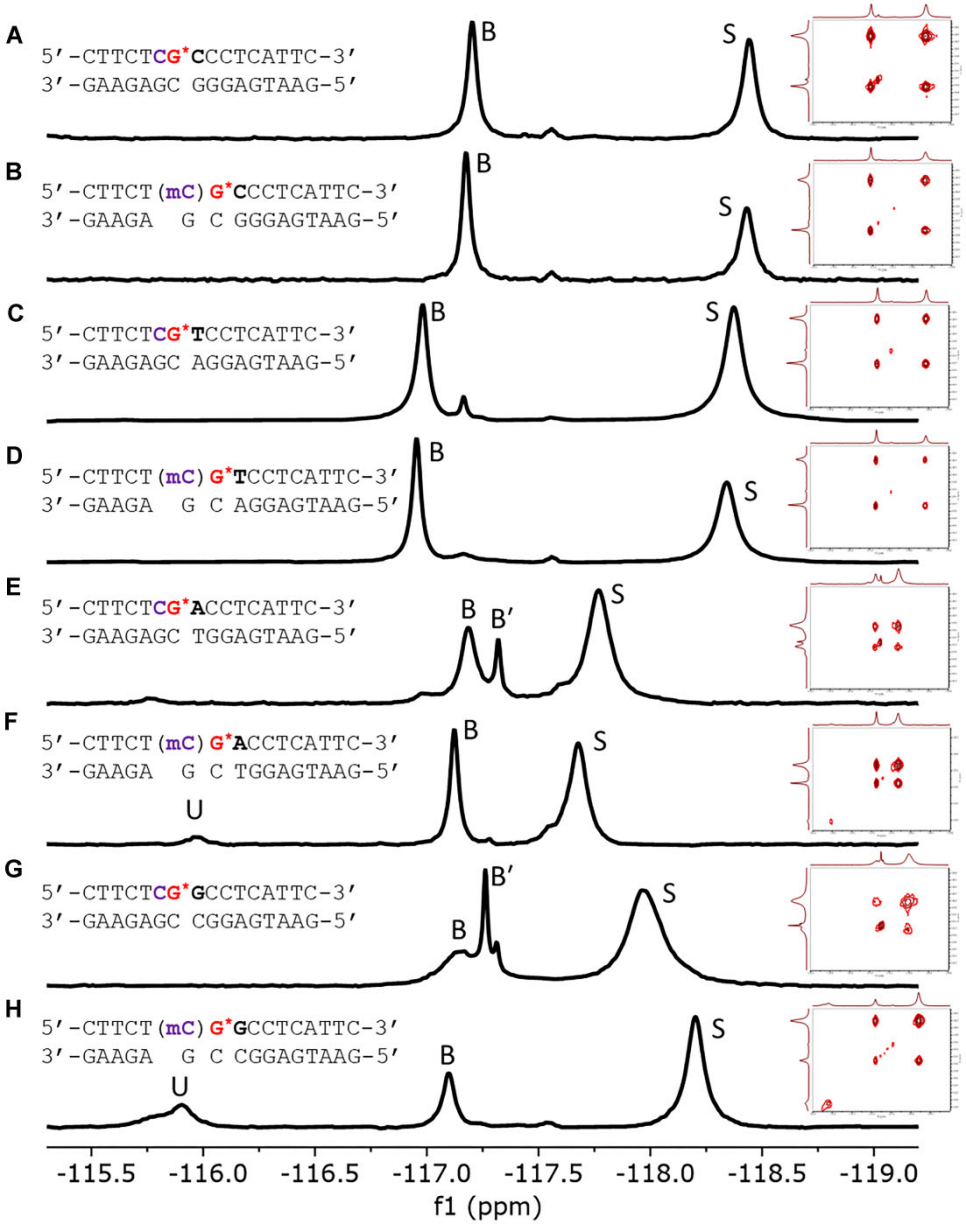
Stacked 1D ^19^F NMR and corresponding EXSY spectra (insets) at 25°C of the FAF-modified (G*) 16mer duplexes showing relative B/S heterogeneity. The B and B-like (B′) conformers appear as downfield signals around −117 ppm and the S conformer appears as a more upfield signal around −118 ppm. The uncharacterized conformation of unknown structure signal is denoted by U. From top to bottom: (**A**) -CG*C-; (**B**) -mCG*C-; (**C**) -CG*T-; (**D**) -mCG*T-; (**E**) -CG*A-; (**F**) -mCG*A-; (**G**) -CG*G-; and (**H**) -mCG*G-. See Table 1 for observed B/S population ratios.

^19^F NMR was used to probe the lesion-induced conformational heterogeneity of DNA duplexes containing dG-FAF in normal or epigenetic sequence contexts. It was found that dG-FAF adopts a mixture of two major conformations in these sequence contexts (Figure 2), B (−116.75 and −117.50 ppm) and S (−117.50 and −118.75 ppm), which can be assigned based on shielding patterns, relative chemical shifts and deuterium isotope effects (i.e. solvent exposure of the fluorine nucleus) (10). Population ratios of the major conformations were evaluated based on integration of the ^19^F signals in the 1D spectra for each sequence (Table 1). Interestingly, the -CG*A- (Figure 2E) and -CG*G- (Figure 2G) contexts exhibited a significant population of one or more secondary, B-like conformations (designated B′) when the 5′ flanking base was C but not 5mC (Figure 2F and H). Furthermore, sequences with a 5′ flanking 5mC and a 3′ flanking purine [-mCG*A- (Figure 2F) or -mCG*G- (Figure 2H)] exhibited a third downfield signal in the 1D spectra between −115.50 and −116.25 ppm, denoted by U (uncharacterized conformation of unknown structure).

**Table 1.** Conformational ratios of the dG-FAF lesion in 16mer duplexes based on integration of signals from 1D ^19^F NMR

	Conformation^a^^,^^b^			
Sequence	B (%)^c^	S (%)	U (%)^d^	Bypass (%)^e^
-CG*C-	41	59		55
-mCG*C-	46	54		80
-CG*T-	47	53		55
-mCG*T-	53	47		75
-CG*A-	36	64		121
-mCG*A-	35	60	5	48
-CG*G-	37	63		123
-mCG*G-	22	60	18	42

^a^Population ratio generated from integration of simulated 1D ^19^F NMR spectra.

^b^Standard deviation <1%.

^c^Sum of B and B′ conformations.

^d^Uncharacterized conformation of unknown structure. Conformations with <1% population have been omitted.

^e^Standard deviation for bypass is between 1.6% and 9.0%. See Figure 5 for details.

EXSY indicated an exchange between the B and S conformations in all sequence contexts (Figure 2, inserts, and Supplementary Figures S4–S7). The U signal in the -mCG*A- and -mCG*G- (Figure 2F and H) 1D NMR spectra was not in exchange with either B or S conformations. This was further confirmed with 1D CEST experiments, where each major NMR signal was selectively saturated, and any signals in exchange with the saturated peak should also saturate. Indeed, when either signal corresponding to the B and S conformations was saturated, the other was saturated, but the downfield signal was not. Similarly, when the downfield signal was saturated, neither signal corresponding to the B and S conformations was saturated (Supplementary Figures S8 and S9). Taken together with the relative chemical shift and the EXSY results, the U signal suggests a deshielded fluorine nucleus, which could indicate an open duplex (probably bulge) around the lesion site, leaving the fluorine nucleus exposed and unable to exchange with the B and S conformations or not readily detectable on an NMR scale in the CEST experiment.

Imino protons in the ^1^H NMR spectra (11.0–14.5 ppm) give further insight into the conformational heterogeneity and the nature of duplex formation (Supplementary Figure S11). More than 16 signals were present at varying intensities in all eight duplexes, indicative of two or more conformations in exchange, consistent with the above mentioned 1D, 2D and ^19^F NMR results. The occurrence of low-intensity upfield signals in the region of 9–11 ppm indicates the exposed guanine imino proton of the FAF-modified dG residue and the presence of non-S/B duplex conformations in the purine-flanked sequences (9,45,46).

### Induced circular dichroism

**Figure 3.**
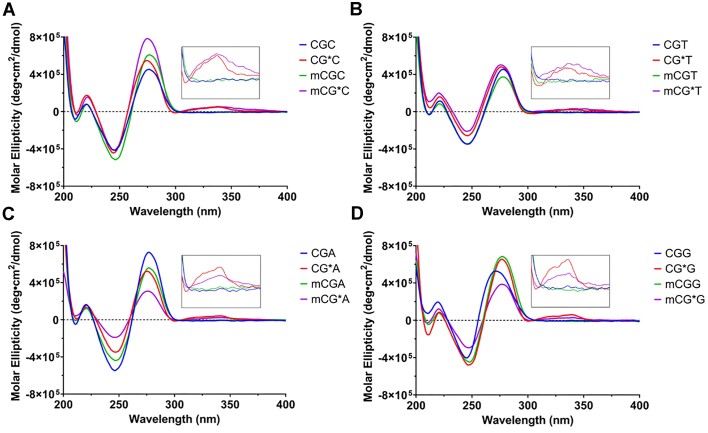
CD spectra of (**A**) unmodified and dG-FAF -CGC- and -mCGC- duplexes; (**B**) unmodified and dG-FAF -CGT- and -mCGT- duplexes; (**C**) unmodified and dG-FAF -CGA- and -mCGA- duplexes; and (**D**) unmodified and dG-FAF -CGG- and -mCGG- duplexes. Insets: ICD effect (295–390 nm) from FAF modification.

Duplex formation of the fully paired duplexes (Figure 3) and at each step of simulated replication (i.e. primer elongation; Supplementary Figures S12–S15) was also studied spectroscopically (2,32). Both control and FAF-modified duplexes showed an S-shaped CD curve with a positive signal at 275 nm and a negative signal at 250 nm, which is characteristic of normal B-DNA (Figure 3) (47). In FAF-modified duplexes, a slight negative ellipticity between 290 and 300 nm and a lesion-induced circular dichroism (ICD) effect (1,32) between 300 and 380 nm were seen, which increased with primer elongation and were not present in the unmodified control duplexes. Modified duplexes were also blueshifted (∼2–3 nm) relative to their unmodified controls, which is a result of bending of the DNA backbone around the lesion (2,18,48). These results are consistent with a conformational mixture. Ellipticity increased with primer elongation opposite the unmodified and FAF-modified templates; however, ellipticities at both 250 and 275 nm of -mCG*A- and -mCG*G- during primer elongation (Supplementary Figures S14D and S15D) and in the fully paired duplexes (Figure 3C and D, purple traces) were generally less well defined than either the corresponding unmodified controls or the unmethylated -CG*A- and -CG*G- duplexes, consistent with less duplex present. The ICD effect was also reduced in these duplexes.

### Thermal and thermodynamic stability

Thermal stability of the duplexes (Supplementary Figure S16) and as a function of primer length (Supplementary Figures S17–S20) was determined by UV melting. Thermal and thermodynamic parameters calculated from UV melting curves are summarized in Supplementary Table S2.

**Figure 4.**
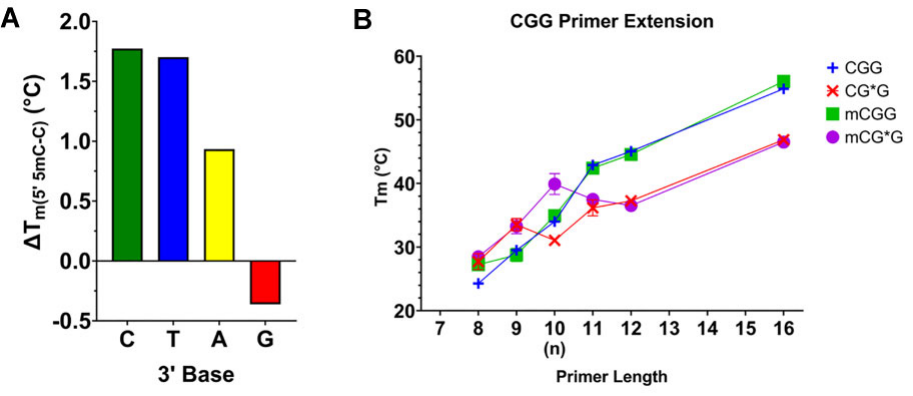
(**A**) Difference in melting temperature (Δ*T*_m_) between the methylated (meCpG) and unmethylated (CpG) duplexes for each (m)CG*N pair. Data presented as the difference arithmetic means (*n* = 5). *X*-axis labels indicate the 3′ base (*N*). (**B**) Melting temperature of the (m)CGG duplexes as a function of primer length. Data presented as the arithmetic mean (*n* = 5) ± standard deviation. Some error bars are too small to be shown.

All duplexes exhibited typical sigmoidal melting curves indicating that they underwent characteristic helix–coil transitions, with *T*_m_ as the midpoint of the transition. Δ*T*_m_ is defined as the difference in melting temperature between either the unmodified control and FAF-modified duplexes or the sequences containing cytosine and 5mC. There were no differences in *T*_m_ between the 5′ C and 5mC for the unmodified duplexes, except for -mCGG- that had a *T*_m_ 1.2°C higher than that of -CGG-. All FAF-modified duplexes were significantly destabilized relative to their corresponding control duplexes as expected, with an average Δ*T*_m,FAF_ = −8.0°C; sequences with greater G:C content around the lesion site (-CGC- and -CGG-) exhibited higher *T*_m_ overall. For sequences with a 3′ flanking pyrimidine (-CG*C- and -CG*T-), the sequences with 5mC were more stable by an average of +1.8°C than when the 5′ base was C. In contrast, when the 3′ flanking base was A, 5mC was only slightly more stable than C (Δ*T*_m,5mC–C_ = +0.9°C), whereas when the 3′ flanking base was G, the *T*_m_ of the meCpG was 0.4°C lower than that of the unmethylated CpG (Figure 4A). The methyl effect on *T*_m_ was statistically significant for all modified duplexes (*P* < 0.000001 for the -CG*C-, -CG*T- and -CG*A- pairs; *P* = 0.0024 for the -CG*G- pair). Interestingly, the -CG*T- and -mCG*T- duplexes (flanked by a T:A base pair on the 3′ side) showed the lowest melting temperatures (38.3 and 40.0°C, respectively), whereas the -CG*A- and -mCG*A- duplexes (flanked by an A:T base pair on the 3′ side) had *T*_m_ values of 42.5 and 43.4°C, respectively. This represents a respective Δ*T*_m_ of 4.2 and 3.4°C between the CpG and meCpG, respectively, for the T:A versus A:T pair, showcasing the effects of base polarity on thermal stability, similar to previously reported trends. Conversely, the differences between C and 5mC for a flanking C:G versus G:C pair were only 1.30 and 0.84°C, respectively, indicating that base polarity is less consequential for these contexts.

*T*_m_ generally increased with primer length from the 8mer (*n* − 2) duplexes to the 12mer (*n* + 2) duplexes, while Δ*H*, Δ*S* and Δ*G* became increasingly negative (Supplementary Table S2), but some interesting sequence-dependent trends were observed. For -CGT- and -mCGT-, *T*_m_ was mostly unchanged for the 8mer (*n* − 2), 9mer (*n* − 1) and 10mer (*n*) duplexes for both the control and FAF-modified sequences. Once the primer was extended past the lesion site, however, the control duplexes became much more thermally stable (Δ*T*_m,U-FAF_ > 10°C), with little difference between C and 5mC (Supplementary Figure S18). For -CGC-, -mCGC-, -CGA-, -mCGA- and -CGG-, the *T*_m_ of the controls steadily increased with primer length, while the FAF-modified duplexes were briefly destabilized at the position opposite the lesion (10mer) (Supplementary Figures S17, S19 and S20). Intriguingly, the -mCG*G- lesion site was 2.4°C more stable than its *n* + 1 duplex, and 3.4°C more stable than its *n* + 2 duplex (Figure 4B). This indicates that the FAF moiety may stack favorably with the 3′ G in the template strand to stabilize the lesion site at the replication fork, but potential steric hindrance from the methyl group and bulging of the duplex from intercalation of the lesion from rotation of the guanine base around the glycosidic bond into the *syn* conformation may compromise hydrogen bonding between the following base pairs. Similarly, Δ*H* and Δ*S* were abnormally higher (more positive) at the *n* + 1 (11mer) position in the -mCG*G- duplex (Supplementary Table S2), further reinforcing the hypothesis of a destabilized conformation.

### Lesion bypass and mutagenicity in cell

After characterizing the biophysical properties of the dG-FAF adduct under epigenetically relevant conditions, we studied the biological properties of the adduct *in cell*, particularly its effects on DNA replication (i.e. replication efficiency/bypass and mutagenicity compared to unmodified dG). We used a vector technique developed and validated by the Essigmann lab. The method entails the CRAB assay for testing replication bypass/block and the REAP assay for mutagenicity (29–31). A single-stranded M13 DNA vector containing the dG-FAF adduct was replicated in *E. coli* containing an inactive AlkB mutant protein (AlkB^−^/HK82).

There were several reasons to choose the *E. coli* system to demonstrate the clear picture of the dG-AF adduct’s biological properties in cell; first, a single-stranded DNA vector was chosen to avoid repair of the bulky dG-AF adduct that would be recognized by NER proteins such as XPC in specific secondary structures, namely the single strand–double strand DNA junction (49–51). Thus, the cellular data reflected the biological properties of dG-AF adduct without complications from NER repair. Second, the reason to choose the AlkB^−^ (HK82) genetic background was to avoid modification of 5mC by AlkB, even though the dG-AF adduct is not a substrate of AlkB. 5mC has been reported to be oxidatively modified by the TET family enzymes (TET1–3), AlkB and its human homologs ABH2 and ABH3 to 5-hydroxymethylcytosine, 5-formylcytosine and 5-carboxylcytosine (52–54). Other AlkB homologs could also potentially modify 5mC (55). Using human cells with all those proteins knocked out would be difficult. To simplify those complications, we selected the *E. coli* system because there is only one AlkB protein and no TET proteins. Also, the AlkB^−^ cell showed the expected replication block and mutation patterns of eA (Supplementary Figure S31), which was used as a positive control to validate the successful application of the CRAB and REAP methods (30). Finally, certain DNA adducts have been studied by vector systems in both *E. coli* and human cells; the biological properties (replication block and mutagenicity) generated from the two sources are very similar (56,57). Those observations validated the usage of *E. coli* cells as a model system, even though 5mC does not exist in *E. coli*, to study certain DNA adducts, such as dG-AF studied in this paper.

The dG-FAF lesion was not significantly mutagenic (average G incorporation >97% in the REAP assays) in the sequence contexts examined (Supplementary Figure S21). The sequence contexts with the highest rate of mutations were -CG*A- and -CG*G-, which also exhibited the most efficient bypass. The frequency and types of mutations are consistent with previously reported results (37).

**Figure 5.**
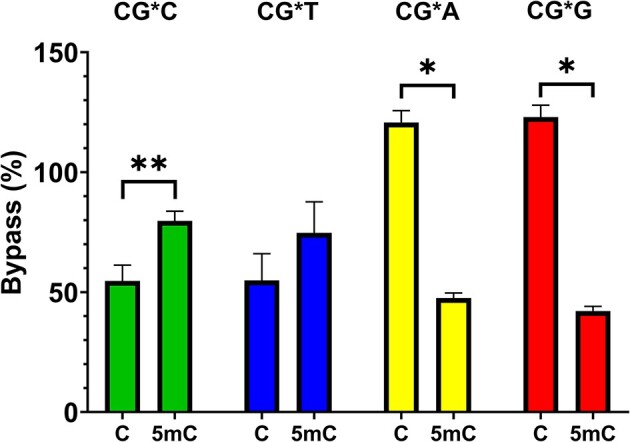
Bypass of dG-FAF in HK82 *E. coli* (AlkB^−^). Data presented as the arithmetic mean (*n* = 3) ± standard deviation. G* = dG-FAF. Horizontal axis labels denote 5′-C or 5′-5mC. Statistical significance: *P < 0.05 and **P < 0.01. *Note*: Bypass >100% is common as bypass in the CRAB assay was calculated relative to the identical sequence containing an unmodified G (58,59).

The lesion bypass (CRAB) assay revealed substantial sequence- and conformation-dependent bypass differences (Table 1 and Figure 5). In sequence contexts with a 3′ pyrimidine, the meCpG duplex was bypassed more efficiently than the corresponding CpG sequence (55% versus 78%, respectively, on average; *P*< 0.01). Bypass seemed to be inversely correlated with population of the S conformer in these sequences (Figure 2 and Table 1). Conversely, sequence contexts where a 3′ purine flanked the lesion site exhibited significantly less bypass on average when the 5′ base was 5mC relative to C (45% versus 122%, respectively; *P*< 0.05). These results were statistically significant for all but the -CG*T- pair (*P* = 0.11). Of note, we observed >100% bypass for the mCG*A and mCG*G sequences; this was not uncommon for certain lesions as the bypass efficiency is calculated relative to the same sequence containing an unmodified G and has been documented in the literature. For example, Essigmann and coworkers observed that 3-methylcytosine has a bypass efficiency of 115.2% in the DinB^−^*E. coli* cell (58). Another example is that Wu *et al.* observed that an alkyl phosphotriester lesion (Sp-Et-PTE) had a bypass efficiency of >300% compared to a non-lesion counterpart (59). Moreover, the -mCG*A- and -mCG*G- sequences exhibited significant populations of the U conformation (uncharacterized bulge-type duplex), lending one possible explanation for their decreased bypass compared to other sequences. Overall, the bypass assay results correlate well with the conformation distribution obtained from the biophysical studies in the CG*C and CG*T sequences, but the relationship between conformation and bypass in the CG*A and CG*G cases is more complex. Taken together with the mutagenesis assay, dG-FAF is more likely to cause toxicity by blocking replication rather than being mutagenic.

## Discussion

DNA sequence context is a strong determinant of the conformational aspects and subsequently the mutational and repair outcomes of bulky dG lesions such as AF, AAF, ABP and BPDE (1–3,5). During replication, the damage may either block replication or be bypassed in an accurate or mutagenic manner. Most research has focused on the mutagenic end results of bulky lesions in epigenetic sequence contexts with little attention to conformation or the intermediate, dynamic steps of replication bypass/block. Most 5mC cellular studies also focus on a specific sequence (e.g. p53). Although cytosine methylation does not significantly affect the structure or replication of undamaged DNA, it is not well understood how 5mC influences conformation in the immediate sequence context containing a bulky lesion. The present study focused on bypass as an outcome to correlate with lesion-induced conformational changes in sequences containing a meCpG site.

### Effects of 5mC on sequence-dependent conformational heterogeneity and duplex stability

The B/S conformational ratios generated from the NMR results are in line with the meCpG-induced conformational switch to an intercalated structure seen with the *anti*-BPDE adduct reported by Zhang *et al.* (28). Planar bulky lesions such as dG-FAF show larger populations of the stacked conformer relative to ‘twisted’ lesions such as dG-FABP (which is structurally similar to dG-AF but has more rotational freedom as it lacks the methylene bridge between the aromatic rings) (10,43). Stacking is reinforced by the expanded aromatic ring system and enhanced π-stacking capabilities when the 3′ base is a purine, as reported in Meneni *et al.*, where the population of the S conformer decreased in the order of 3′-G > A > C > T for dG-FAF (43). This was illustrated by Cai *et al.*, where the dG-FAF lesion adopted a 25%:75% B:S ratio in the -TG*GT- sequence context when the lesion was on G_1_ (3′ purine), but only 17% S when the lesion was on G_2_ (-TGG*T-) and flanked by a pyrimidine that contains only a single ring. Conversely, the less rigid dG-FABP exhibited a much lower contribution from the S conformer (67%:33% B:S ratio) on G_1_ in the same sequence, and no S (100% B) on G_2_ (2). Our NMR studies showed that the -CG*A- and -CG*G- duplexes adopted a majority of the S conformer, 64% and 63%, respectively.

As mentioned earlier, 5mC has been shown to increase the thermal stability of the modified duplex (26,27). Our data agree with this finding, as evidenced by the elevated *T*_m_ of the -mCG*C-, -mCG*T- and -mCG*A- duplexes compared to their respective unmethylated partner sequences (Figure 4A). The only exception to this trend was -mCG*G-, which was 0.4°C less stable than its unmethylated counterpart, but exhibited 18% U conformation. If this conformation is a bulge or bubble around the lesion site, this would explain the decreased thermal stability, as it would destabilize the duplex. While -mCG*A- also revealed a small percentage (5%) of this suspected bulge conformation, it was still more stable than -CG*A-, but only to half the extent of the -CG*C- and -CG*T- sequences.

The additional conformation induced by 5mC in the purine-flanked sequences and heterogeneity in the upfield region of the imino ^1^H NMR spectra pose an interesting conformational perspective. While 5mC is not thought to be structurally disruptive in unmodified DNA, this small steric addition may have significant effects on duplex formation in damaged DNA. While there was little difference between C and 5mC in the CD spectra of the -CG*C- and -CG*T- pairs, striking methyl effects were seen in the -CG*A- and -CG*G- sets (Figure 3C and D and Supplementary Figures S14D and S15D). The -mCG*A- and -mCG*G- sequences exhibited significantly smaller ellipticities throughout primer elongation and in the fully paired duplexes, indicative of decreased duplex formation. More evidence of this is that the -mCG*G- duplex was both thermally and thermodynamically weaker at the *n* + 1 and *n* + 2 positions than the lesion position (*n*) during primer elongation. In contrast, all other modified duplexes were weakened at the position opposite the lesion and became increasingly stable with continued primer elongation (Figure 4B and Supplementary Table S2). Previous molecular modeling studies have shown that in a FAF-modified 12mer duplex with a similar sequence to the templates used herein, the 3′ G:C base pair in the -AG*G- context was broken, but the base pairing in the -AG*T- context more resembled that of undamaged control duplexes (43). The B conformer is a thermodynamic stabilizer, while the S conformer is thermodynamically destabilizing in the fully paired duplex; however, lesion stacking stabilizes the single strand–double strand junction at the replication fork (2). This may explain the unusually high melting temperature at the lesion site in -mCG*G-, where the 3′ flanking G provides stabilizing stacking interactions with the planar FAF, but once the primer is extended past the lesion site, it may become unstable; the growing complementary strand may not make sufficient contact to hydrogen bond with the template strand. We hypothesize that this is induced by steric hindrance from the methyl group and the widening of the duplex from the large population of the *syn*-S conformer.

### Conformation-dependent lesion bypass and mutagenicity

The dG-AF lesion is known to be weakly mutagenic, unlike some of its bulkier derivatives, such as AAF, in which substituents at the central nitrogen contribute additional steric bulk and promote mutations (11,16,17,19,37). Despite a reported 50% increase in mutations from lesions such as 1-aminopyrene in cells (24), 5mC did not significantly alter the mutagenicity of dG-FAF in the sequence contexts tested herein (Supplementary Figure S21). The mutagenicity of m5C is relatively weak compared to the unmodified cytosine (60).

In both pairs of pyrimidine-flanked sequences, the meCpG was more efficiently bypassed (75–80%) than the unmethylated CpG (55%), concurrent with the reduction in the S conformer (Figure 5). This is likely attributable to the fact that while in the S conformation the glycosidic bond of the modified guanine is rotated into the *syn* position relative to the 2′-deoxyribose sugar, which breaks the Watson–Crick base pairing, causing a slight widening (bulge) in the duplex, as opposed to the B conformation in which the guanine remains *anti* to the sugar, leaving the Watson–Crick base pairing intact and maintaining the standard B-form DNA duplex (Figure 1). This S-induced distortion may be too disruptive for a replicative polymerase to accommodate, and it consequently falls off the duplex, stalling replication. In addition to the lesion potentially physically blocking the incoming dNTP during replication, with the guanine in the *syn* glycosidic conformation, the imino protons face away from the incoming dNTPs, lessening their ability to base pair with the complementary strand, leading to reduced bypass.

The population of the S conformer was similarly reduced by 3% in both the -mCG*A- and -mCG*G- sequences relative to their unmethylated partner sequences, but in contrast to the -CG*T- and -CG*C- pairs, they were less efficiently bypassed. This may be attributable to both a reduction in the stabilizing B conformer and the appearance of the U conformation (5% in -CG*A- and 18% in -CG*G-), represented by a downfield ^19^F NMR signal between −115.5 and −116.0 ppm (Figure 2), which did not exchange with either the B or S conformations in EXSY (Supplementary Figures S4–S7) and CEST (Supplementary Figures S8–S10) experiments. As previously noted, this could indicate an open or bulged DNA duplex in which the fluorine is exposed and deshielded. In this case, U conformation would be even more disruptive and destabilizing to the DNA duplex than the S conformer; consequently, more disruption would likely yield reduced bypass, which is what we observed in the -CG*A- and -CG*G- duplexes. Although probably due to a different conformational reason than in the -CG*T- and -CG*C- cases, upon encountering these bulge structures in the -mCG*A- and -mCG*G- duplexes, the result would be similar: the polymerase could not accommodate the significant distortion, would stall and fall off, explaining the reduced lesion bypass. Further studies are necessary to characterize the U conformation; however, it seems to be a highly disruptive conformation that modulates the bypass efficiency of the lesion, particularly in the meCpG context. Additionally, one caveat that should be addressed is that since this is the first report to combine biophysical studies with in-cell bypass and mutagenicity assays, there is no benchmark for the population of U that induces changes in bypass; this is an area that requires further exploration and necessitates similar comprehensive studies of different bulky lesions.

## Conclusions

We have presented a novel conformation–function relationship of the dG-FAF lesion in all possible epigenetically relevant C^#^G*N sequence contexts by selectively methylating the cytosine 5′ to the lesion and systematically varying the 3′ flanking base in a 16mer template sequence. We found that meCpG sites can alter the S/B conformational ratios and thermodynamic characteristics of bulky arylamine lesions, dependent on the identity of the 3′ flanking base, leading to substantial alterations in the replicative bypass of the adduct *in cell*. The present work is the first report that combines biophysical and cellular studies for a comprehensive structure–function investigation and provides a conformational basis for cellular bypass of bulky DNA adducts.

While 5mC is weakly mutagenic and does not block replication on its own (60), its effect on bypass and mutagenicity of neighboring bases is significant and warrants further investigation to confirm the structural causes. Bulky DNA damage (e.g. intrastrand cross-links, arylamines, etc.) is repaired by the NER pathway, which recognizes damage through distortion/destabilization of the DNA duplex (61,62). NER is, in turn, altered by a lesion’s structural and thermodynamic properties rather than its chemical identity; lesions that adopt more disruptive conformations are more likely to be recognized and repaired by NER machinery than less disruptive conformations (3,43,48). Therefore, these highly disruptive conformations may be better recognized and repaired; future studies will include further characterization of the U conformation, probing the downstream effects of this conformational heterogeneity in NER, and long-range 5mC effects.

## Supplementary Material

gkad1038_Supplemental_File

## Data Availability

The data underlying this article are available in the article and in its online supplementary material.
