# Supplementary material for: Conformation-dependent lesion bypass of bulky arylamine-dG adducts generated from 2-nitrofluorene in epigenetic sequence contexts
Source: Nucleic Acids Res. 2023 Nov 11;51(22):12043–53. doi: 10.1093/nar/gkad1038 (PMC10711442; doi:10.1093/nar/gkad1038)
Supplement: gkad1038_Supplemental_File [file gkad1038_supplemental_file.pdf]

**Supporting Information**

Conformation-dependent lesion bypass of bulky arylamine-dG adducts generated from 2-nitroofluorene in epigenetic sequence contexts

Alicia M. Crisalli, Yi-Tzai Chen, Ang Cai, Deyu Li*, Bongsup P. Cho*

Department of Biomedical and Pharmaceutical Sciences, College of Pharmacy, University of Rhode Island, Kingston, RI 02881

## Table of Contents

S3. Supplemental Methods
S6. **Fig. S1.** HPLC purity check of the 8 FAF-modified (G*) single-strand 16mer template sequences.
S7. **Fig. S2.** Separation of dG-FAF monoadducts from unmodified controls.
S8. **Fig. S3.** MALDI-TOF results of SVP-digested (3'→5') oligonucleotides.
S9. **Fig. S4.** The 600 MHz $^{19}$F 2D EXSY spectra of (A) the 16mer CG*C duplex and (B) the 16mer mCG*C duplex.
S10. **Fig. S5.** The 600 MHz $^{19}$F 2D EXSY spectra of (A) the 16mer CG*T duplex and (B) the 16mer mCG*T duplex.
S11. **Fig. S6.** The 600 MHz $^{19}$F 2D EXSY spectra of (A) the 16mer CG*A duplex and (B) the 16mer mCG*A duplex
S12. **Fig. S7.** The 600 MHz $^{19}$F 2D EXSY spectra of (A) the 16mer CG*G duplex and (B) the 16mer mCG*G duplex
S13. **Fig. S8.** 600 MHz $^{19}$F 1D CEST experiments for 16mer duplex mCG*A.
S14. **Fig. S9.** 600 MHz $^{19}$F 1D CEST experiments for 16mer duplex mCG*G.
S15. **Fig. S10.** 600 MHz $^{19}$F 1D CEST experiments for 16mer duplex CG*G.
S16. **Fig. S11.** Stacked imino proton ($^1$H NMR, 9-15 ppm) spectra taken of the FAF-modified 16mer duplexes in 90% H2O/10% D2O.
S17. **Fig. S12.** Overlaid CD spectra taken at 25°C showing the effect of primer elongation opposite CGC series templates.
S18. **Fig. S13.** Overlaid CD spectra taken at 25°C showing the effect of primer elongation opposite CGT series templates.
S19. **Fig. S14.** Overlaid CD spectra taken at 25°C showing the effect of primer elongation opposite CGA series templates.
S20. **Fig. S15.** Overlaid CD spectra taken at 25°C showing the effect of primer elongation opposite CGG series templates.
S21. **Fig. S16.** Melting temperature (Tm) of the 16mer duplexes.
S22. **Fig. S17.** UV melting temperature (Tm) of CGC series duplexes.
S23. **Fig. S18.** UV melting temperature (Tm) of CGT series duplexes.
S24. **Fig. S19.** UV melting temperature (Tm) of CGA series duplexes.
S25. **Fig. S20.** UV melting temperature (Tm) of CGG series duplexes.

S26. **Fig. S21.** Lesion mutational specificity and frequency in HK82 E. coli (AlkB-).
S27. **Fig. S22.** ESI-TOF analysis of lesion-containing 16mer oligos.
S28. **Fig. S23.** Diagram of construction of 58mer lesion-containing oligonucleotide.
S29. **Fig. S24.** Denaturing urea polyacrylamide gel of 58mer lesion containing oligonucleotide.
S30. **Fig. S25.** Diagram of LC-TOF-MS identification of the digestion product from the 58mer lesion-containing oligonucleotide using the CG*C sequence as an illustration (G*=dG-C8-FAF).
S31. **Fig. S26.** ESI-TOF analysis of the digestion products from the 58mer oligos.
S32. **Fig. S27.** Diagram of PCR amiplification for lesion-containing M13 genome.
S33. **Fig. S28.** Diagram of polyacrylamide gel of PCR of lesion-containing M13 genome.
S34. **Fig. S29.** Diagram of the REAP & CRAB procedures.
S35. **Fig. S30.** A typical LC-TOF-MS spectrum of the REAP and CRAB samples.
S36. **Fig. S31.** Mutagenesis and bypass of eA in E. coli.
S37. **Table S1.** Calculated monoisotopic mass and actual m/z measured by MALDI-TOF.
S38. **Table S2.** Thermal and thermodynamic parameters of FAF-modified duplexes.
S40. **Table S3.** List of oligonucleotide and primer sequences (5′→3′) used for the REAP and CRAB assays.
S42. **Table S4.** Calculated and observed monoisotopic MW and m/z value of modified oligonucleotides.
S43. **Table S5.** Calculated and observed monoisotopic MW and m/z value of modified oligonucleotides after digestion.
S44. Supplemental References

**Supplemental Methods**

*Preparation, Purification, and Characterization of Site-Specifically Modified Oligonucleotides*

Briefly, the DNA-reactive *N*-acetoxy-N-(trifluoroacetyl)-7-fluoro-2-aminofluorene was synthesized via biomimetic activation from 2-fluoro-7-nitrofluorene to *N*-hydroyxyesters as previously described.(1, 2) The activated species was then dissolved to 0.01 mg/µL in absolute ethanol (100 µL) and incubated with ~50 ODs of unmodified 16mer oligonucleotides in 300 µL of 10 mM sodium citrate buffer, pH 6.0 at 37 °C for 24 h. The reaction mixtures were syringe filtered and purified to >97% using reverse-phase high performance liquid chromatography (RP-HPLC) and a Phenomenex Luna C18 column (150 × 10 mm, 5.0 µm) (Phenomenex, Torrance, CA, USA) (**Supplementary Figure S1**). A gradient of 12.5-25% acetonitrile in triethylammonium acetate (TEAA) buffer (0.3 M, pH 4.5-6.0) for 25 minutes was used to separate the unmodified and modified oligos. The lower pH buffer was used to separate mono-adducts in oligos containing more than one G (i.e., -$C^{\#}G^{*}$G-) (**Supplementary Figure S2**), whereas the higher pH buffer was sufficient for separating modified from unmodified oligos in sequences containing only one G (i.e., -$C^{\#}G^{*}$T-, -$C^{\#}G^{*}$C-, -$C^{\#}G^{*}$A-).

Lesion position was confirmed via enzymatic digestion and analyzed on a Shimadzu (Kyoto, Japan) AXIMA Performance matrix-assisted laser desorption-ionization time of flight mass spectrometer (MALDI-TOF/MS) equipped with a 50 Hz nitrogen laser operating in reflectron mode. Oligos (200 pmol) were incubated with snake venom phosphodiesterase I (0.2 units) for up to 10 minutes. Aliquots (1 µL) were removed at various time points and quenched on the MALDI plate by mixing with 1 µL of matrix (50% v/v 3-hydroxypicolinic acid (3-HPA; 150mg/mL) in dihydrogen ammonium citrate (DHAC; 50 mg/mL)). The MALDI-TOF results are shown in **Supplementary Figure S3**. It was determined by the digested molecular weights that, for both CGG and mCGG, peak 1 was the $G_2$ adduct and peak 2 was the desired $G_1$ adduct (**Supplementary Figure S2**).

*$^{19}F$ and $^{1}H$ NMR Parameters*

T1 and T2 were measured to be ~0.5 s and 8-18 ms respectively for both signals using -mCGA-; these values are consistent with previously reported T1 and T2 for the B and S conformers.(3) D1 and AQ were therefore set to be 2.0 s and 0.2048 s respectively for $D1 + AQ = 4 - 5 \times T1$ to ensure full relaxation. Spectra were collected using an average of 25000 scans, a 12500 Hz sweep width, and referenced to external trifluoroacetic acid ($CF_3CO_2H$). EXSY spectra were taken in phase-sensitive mode using a NOESY pulse sequence, spectral width 3333.3, 1666.7 Hz, number of scans 256, and a mixing time of 100 ms. Imino $^{1}H$ NMR spectra were taken over 1024 scans with a sweep width of 6250 Hz. FIDs were processed with an exponential line broadening factor of 5 Hz for 1D $^{19}F$ NMR spectra and 2 Hz for 1D $^{1}H$ NMR spectra.

*In Cell Lesion Bypass and Mutagenesis Assays*

Briefly, the constructed M13 viral genomes were mixed with competitor genomes at a 50:1 ratio and transfected into *E. coli* strains by electroporation. Prior to transfection, HK82 ($AlkB^-$) cells were made electrocompetent.(4) The M13 genomes were extracted from the amplified progeny using QIAprep M13 kit (Qiagen). The lesion region was PCR amplified with assay-specific primers followed by double digestion with XhoI and SphI endonucleases to obtain a DNA fragment (20mer/28mer for the adduct sequence and 23mer for the competitor). Analyses of DNA fragments were performed by LC-ESI-TOF-MS (AB Sciex, ABI4600), and all data represent the mean ± standard deviation (SD) of three independent experiments. Liquid chromatographic separation was achieved by using an Acclaim Polar Advantage II C18 column (2.1 × 250 mm; 3 μm) at a flow rate of 0.15 mL/min. Solvent A was 500 mM 1,1,1,3,3,3-hexafluoro-2-propanol (HFIP) in water, and solvent B was 500 mM HFIP in 50% methanol. A solvent gradient was carried out under the following conditions: 25% of B for 1 min, 25 to 50% of B over 2 min, 50 to 75% of B over 20 min, 75 to 100% of B over 1 min, 100% of B for 10 min, 80 to 25% of B over 1 min, and 25% B over 10 min. LC column oven was set at 35 °C during whole running time. ESI was conducted by using a needle voltage of 4.0 kV in a negative ion mode. A heated capillary was set at

350 °C. The nebulizer gas pressure was 35 psi; the heater gas pressure was 20 psi; the curtain gas pressure was 20 psi; the declustering potential was -200 V; and the collision energy was -5 V. To quantify the lesion bypass, the ratio between the intensities of the 20mer (modified) and 23mer (competitor) fragments was determined and normalized to the ratio obtained from the parallel experiment employing an unmodified "G" control, considered 100% bypass. Mutagenicity was analyzed based on the MS results (**Supplementary Table S5; Supplementary Figure S25**), and mutation fraction was calculated by dividing individual mutated oligo signal by the total oligo signals.

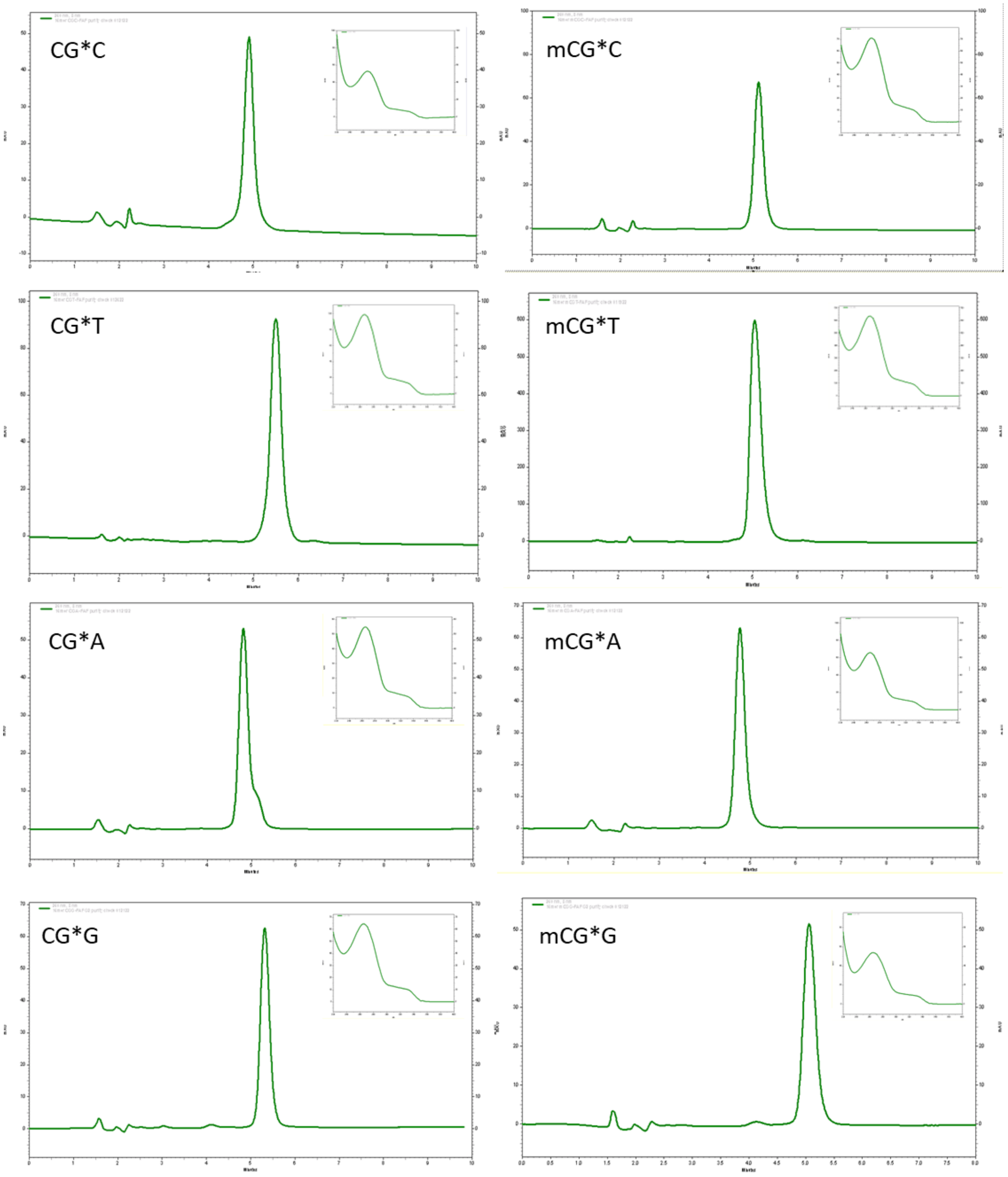


**Fig. S1.** HPLC purity check of the 8 FAF-modified (G*) single-strand 16mer template sequences. Insets: UV max absorption spectra (260 nm) showing a shoulder of FAF-modified 16mer oligonucleotides. **d[5′-CTTCT$\underline{C^{\#}G^{*}\mathit{N}}$CCTCATTC-3′], where $C^{\#}$ is C or 5mC, $G^{*}$ is G or G-FAF, and *N* is A, T, C, or G.**

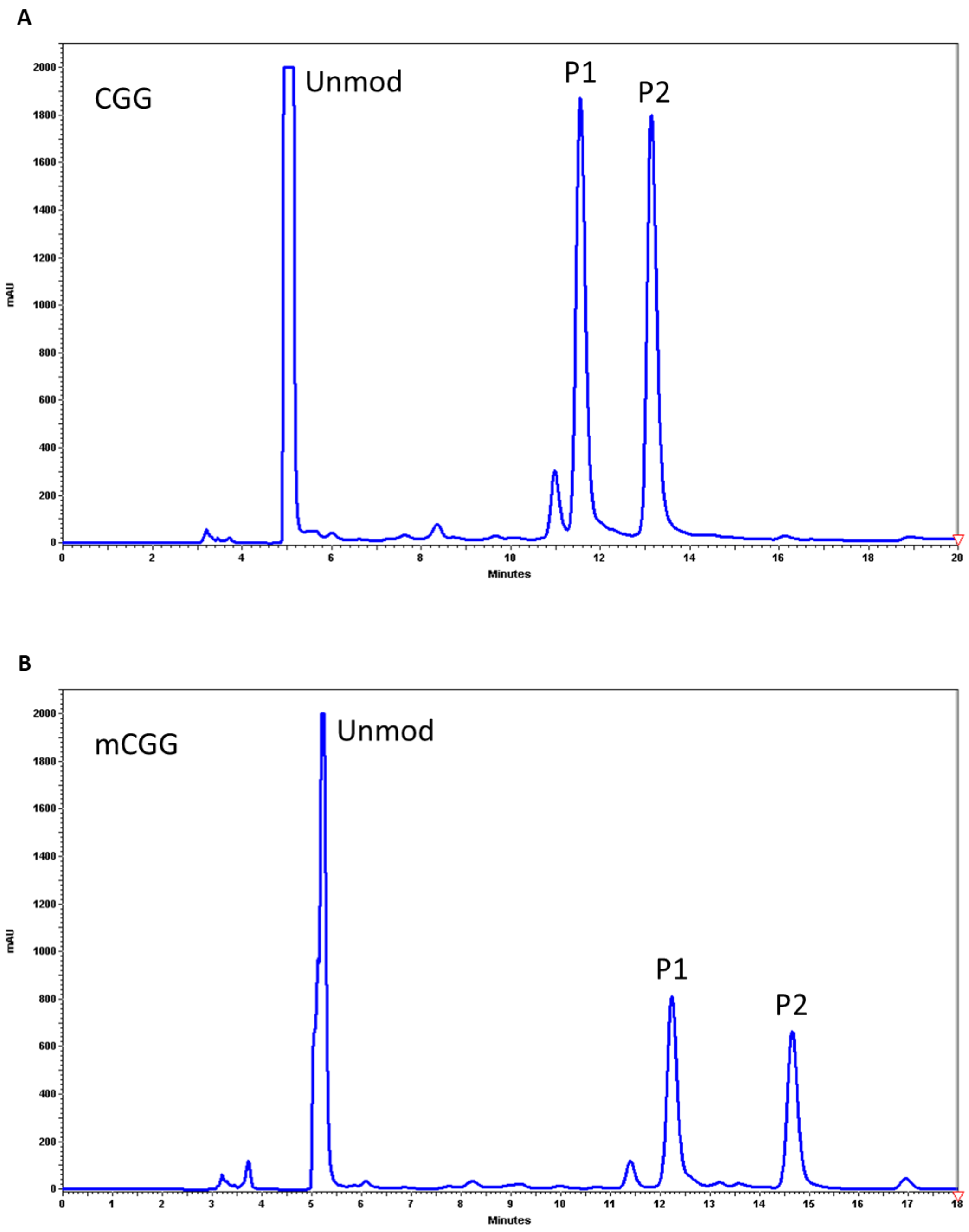


**Fig. S2.** Separation of dG-FAF monoadducts from unmodified controls in (A) single-stranded 16mer -CGG- and (B) single-stranded 16mer mCGG sequences. P1 and P2 represent the monoadducts on G2 and G1 respectively, and assignment of the peaks was determined by 3′▪5′ enzymatic digestion by snake venom phosphodiesterase (SVP) followed by MALDI-TOF analysis (see Table S1 and Figure S3). **d[5′-CTTCT$C^{\#}G^{*}N$CCTCATTC-3′], where $C^{\#}$ is C or 5mC, $G^{*}$ is G or G-FAF, and *N* is A, T, C, or G.**

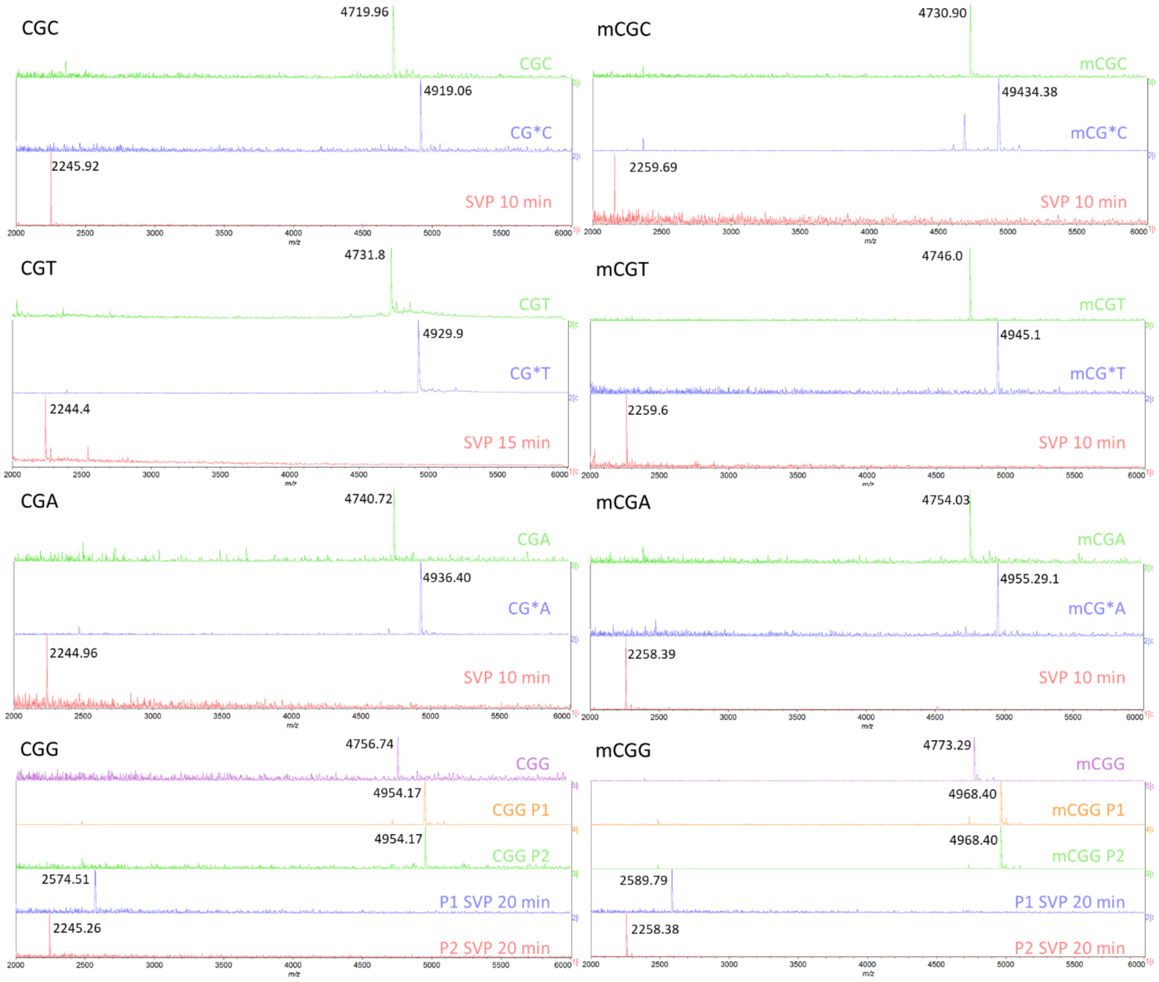


**Fig. S3.** MALDI-TOF results of SVP-digested (3′→5′) oligonucleotides. See Table S1 for calculated monoisotopic masses. **d[5′-CTTCT<u>C$^{\#}$G$^{*}$*N*</u>CCTCATTC-3′], where C$^{\#}$ is C or 5mC, G$^{*}$ is G or G-FAF, and *N* is A, T, C, or G.**

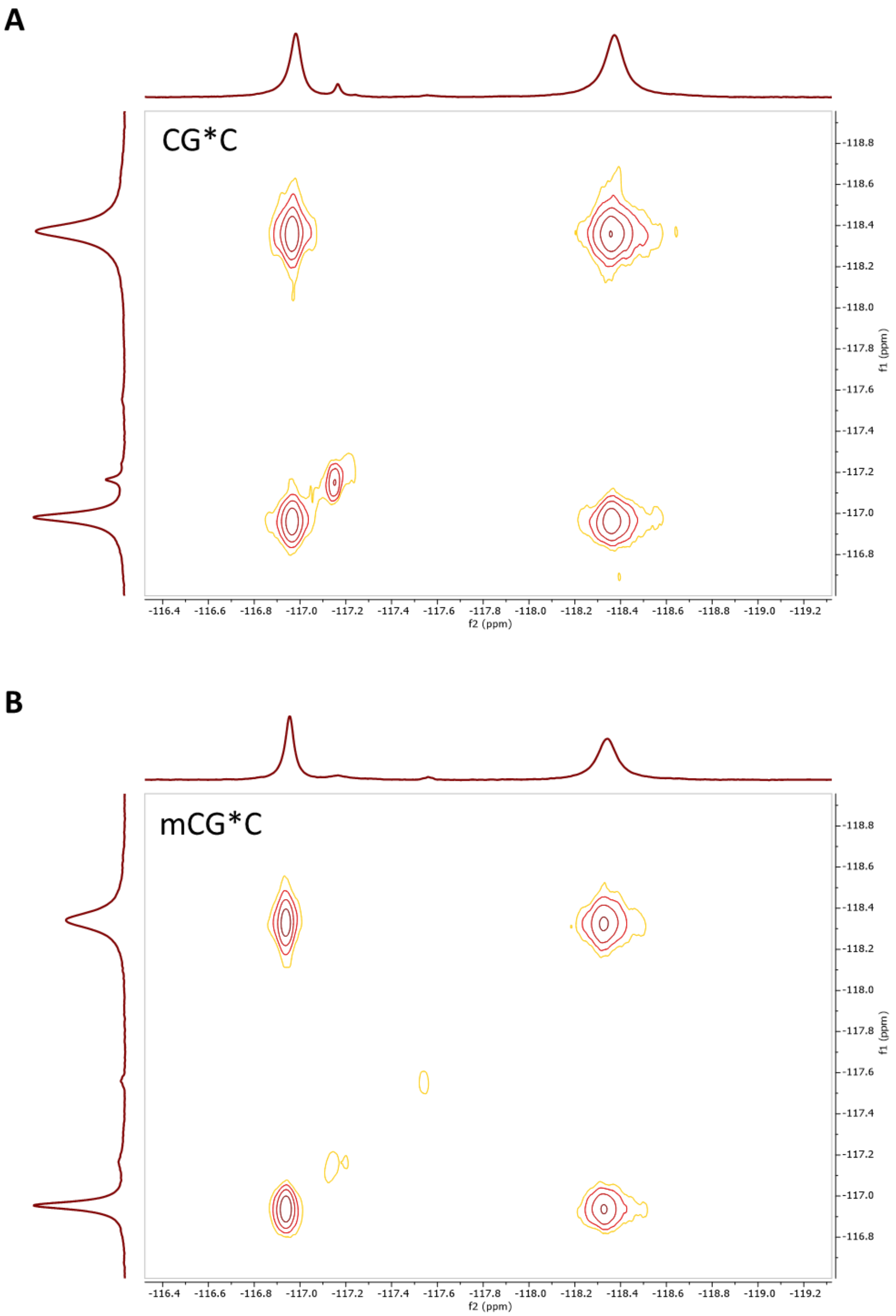


**Figure S4.** The 600 MHz $^{19}$F 2D EXSY spectra of (A) the 16mer CG*C duplex and (B) the 16mer mCG*C duplex.

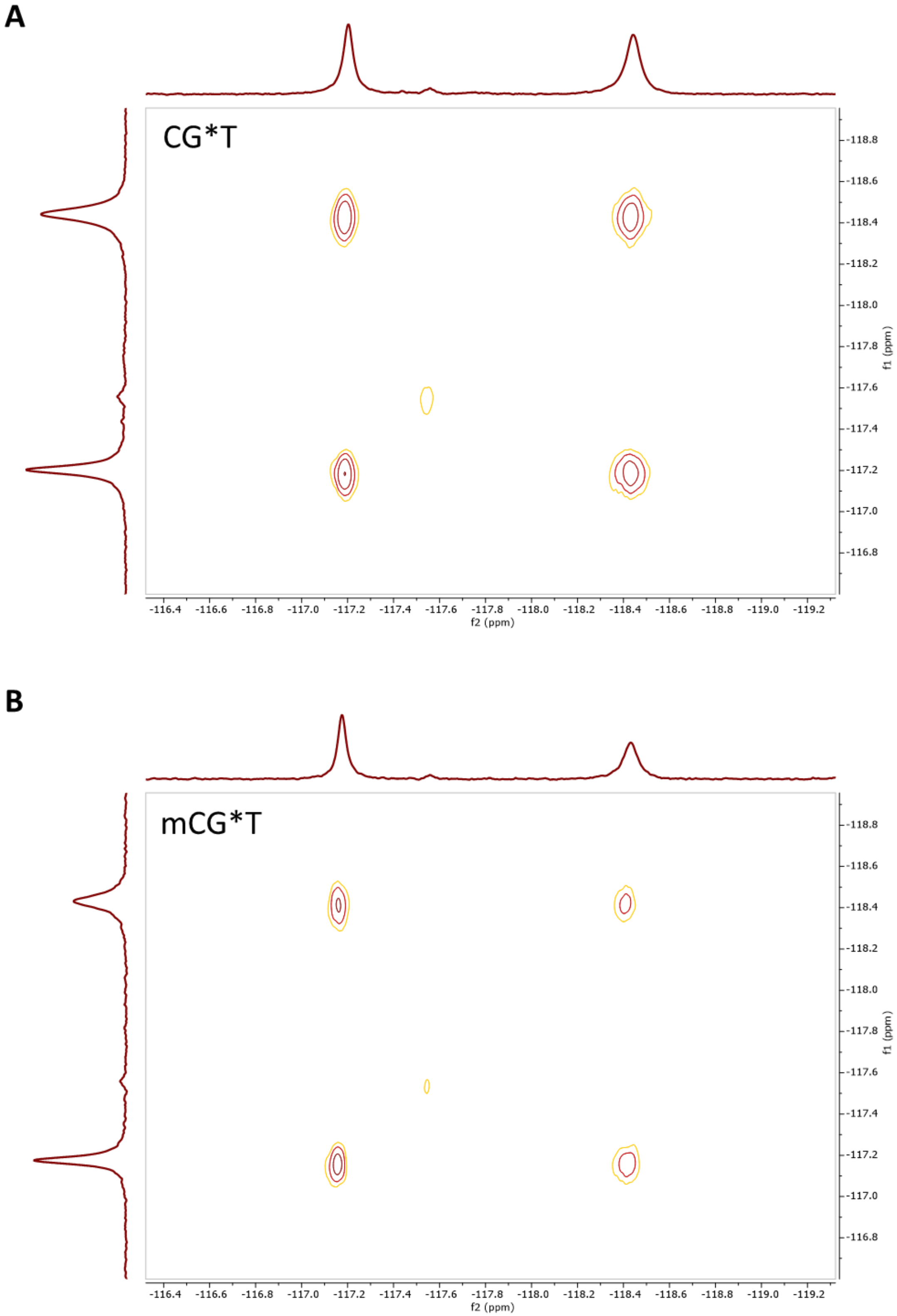


**Figure S5.** The 600 MHz $^{19}F$ 2D EXSY spectra of (A) the 16mer CG*T duplex and (B) the 16mer mCG*T duplex.

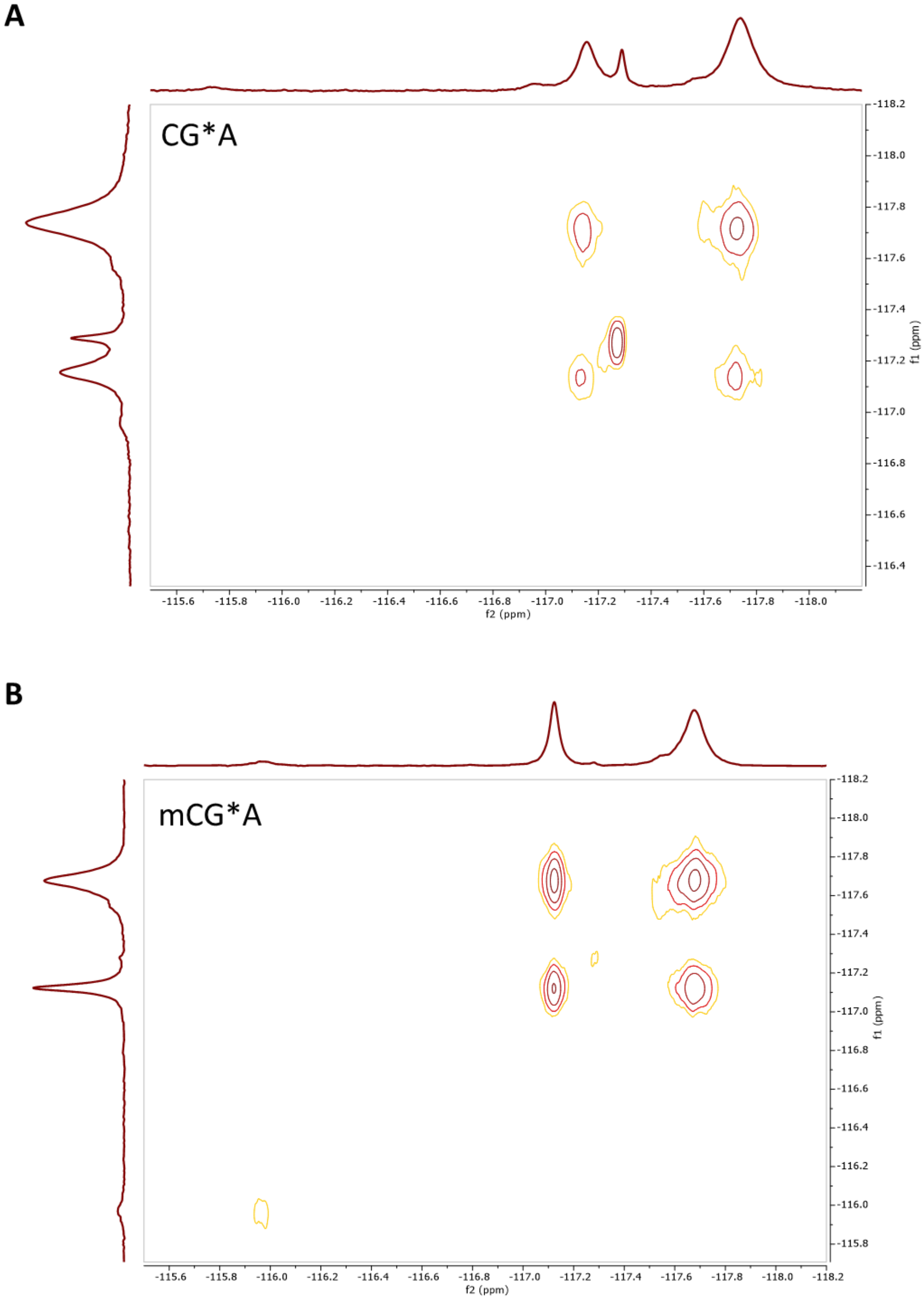


**Figure S6.** The 600 MHz $^{19}$F 2D EXSY spectra of (A) the 16mer CG*A duplex and (B) the 16mer mCG*A duplex.

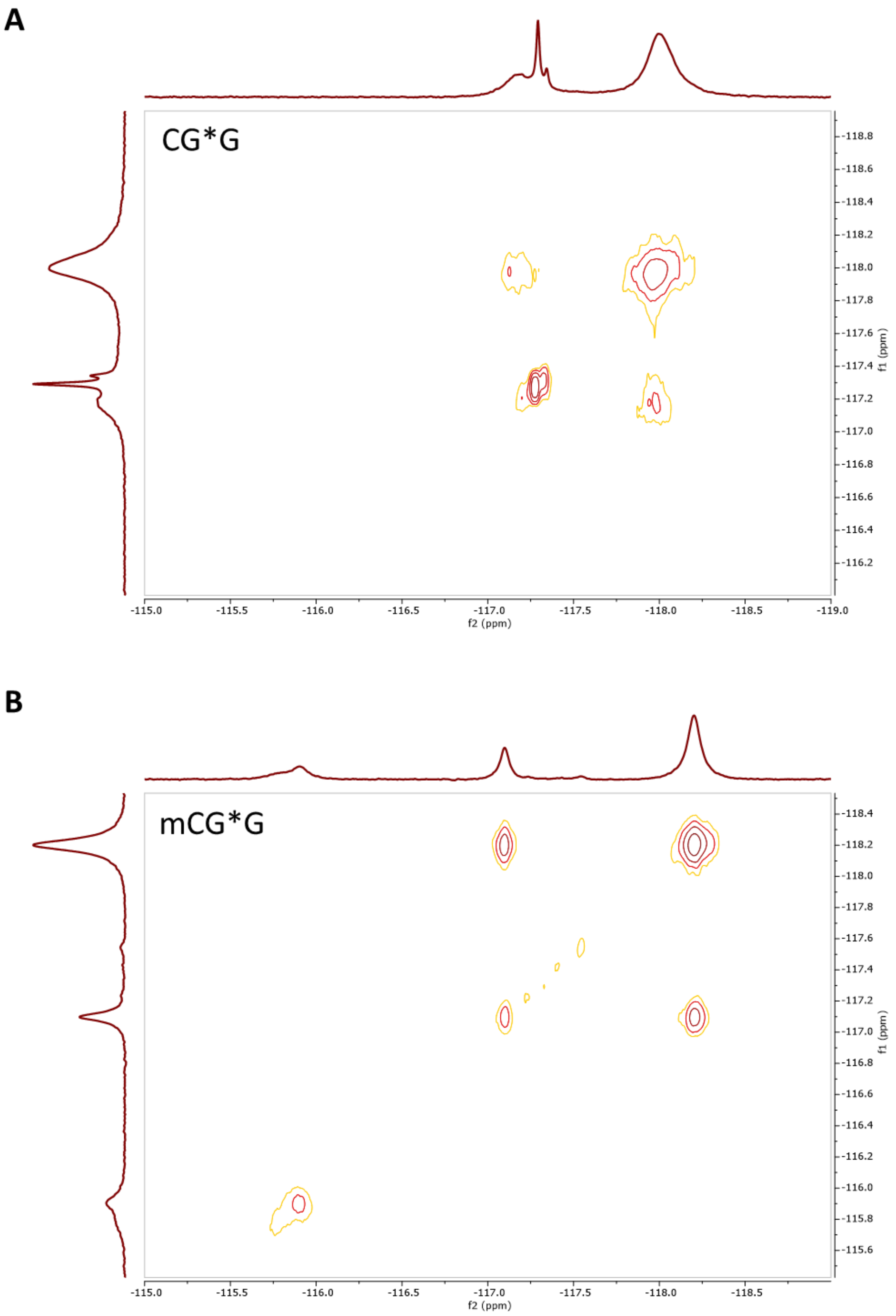


**Figure S7.** The 600 MHz $^{19}F$ 2D EXSY spectra of (A) the 16mer CG*G duplex and (B) the 16mer mCG*G duplex.

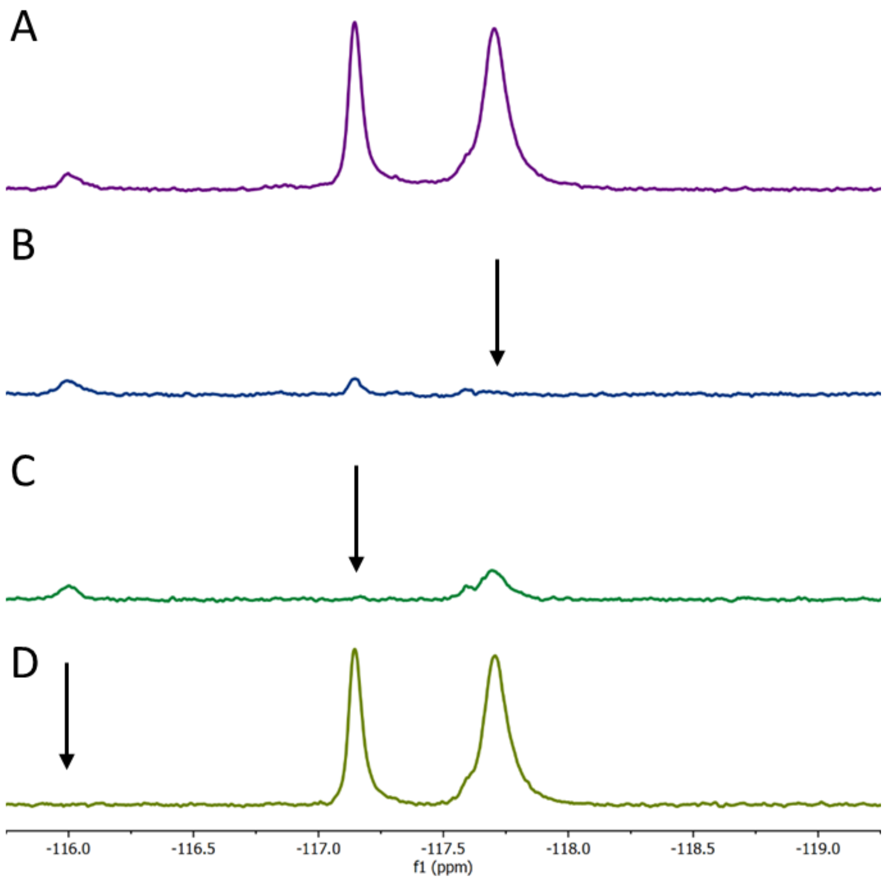


**Figure S8.** 600 MHz $^{19}F$ 1D CEST experiments for 16mer duplex mCG*A. From top to bottom: (A) 1D $^{19}F$ trace (purple); (B) saturation of S signal (-117.7 ppm, blue); (C) saturation of B signal (-117.1 ppm, green trace); (D) saturation of signal at -116.0 ppm (olive trace). Arrows indicate the signal being saturated.

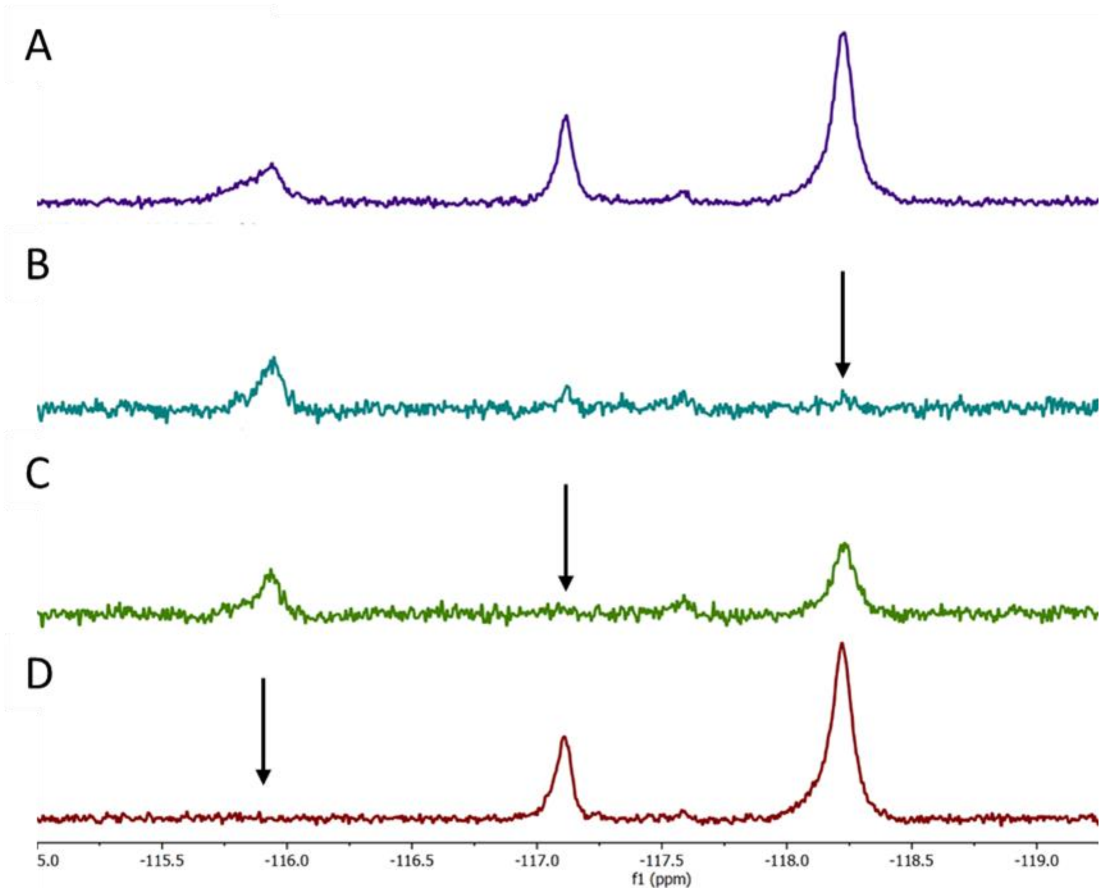


**Figure S9.** 600 MHz $^{19}$F 1D CEST experiments for 16mer duplex mCG*G. From top to bottom: (A) 1D $^{19}$F trace (purple); (B) saturation of S signal (-118.2 ppm, blue); (C) saturation of B signal (-117.1 ppm, green trace); (D) saturation of U signal (-115.9 ppm, red trace). Arrows indicate the signal being saturated.

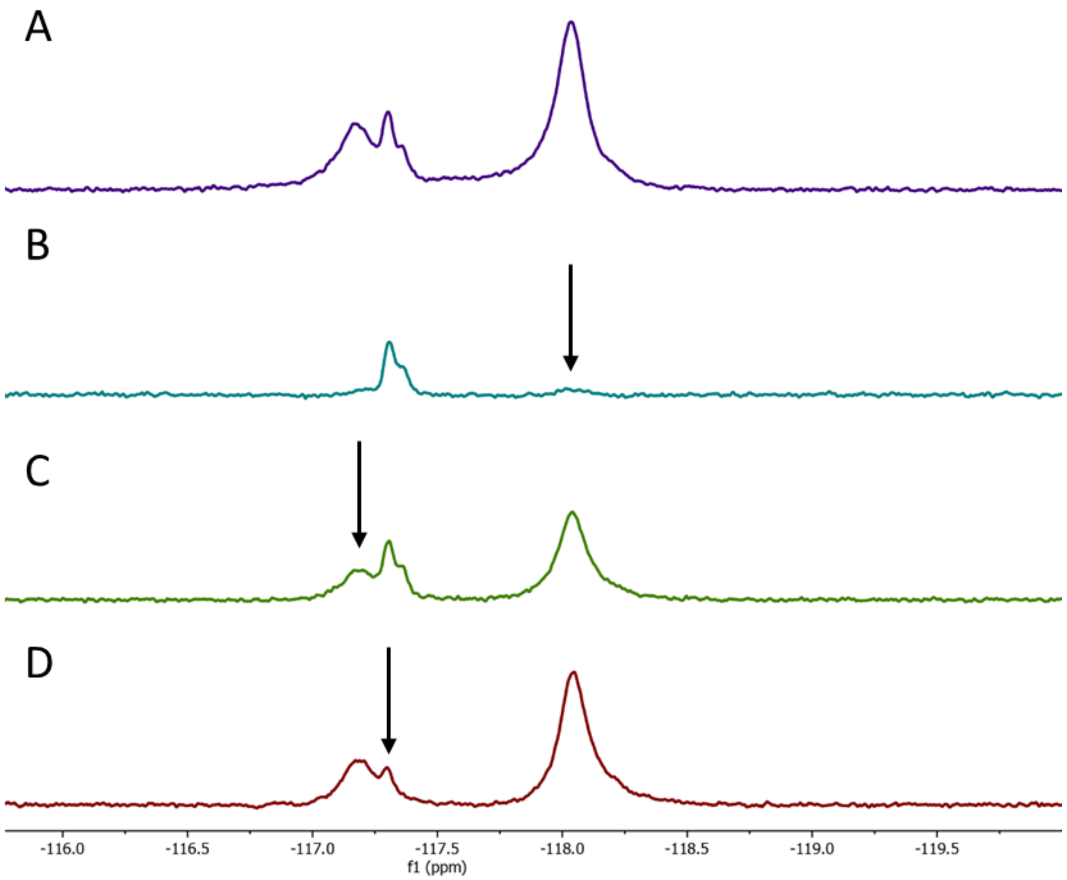


**Figure S10.** 600 MHz $^{19}F$ 1D CEST experiments for 16mer duplex CG*G. From top to bottom: (A) 1D $^{19}F$ trace (purple); (B) saturation of S signal (-118.0 ppm, blue); (C) saturation of B signal (-117.2 ppm, green trace); (D) saturation of B′ signal at -117.3 ppm (red trace). Arrows indicate the signal being saturated.

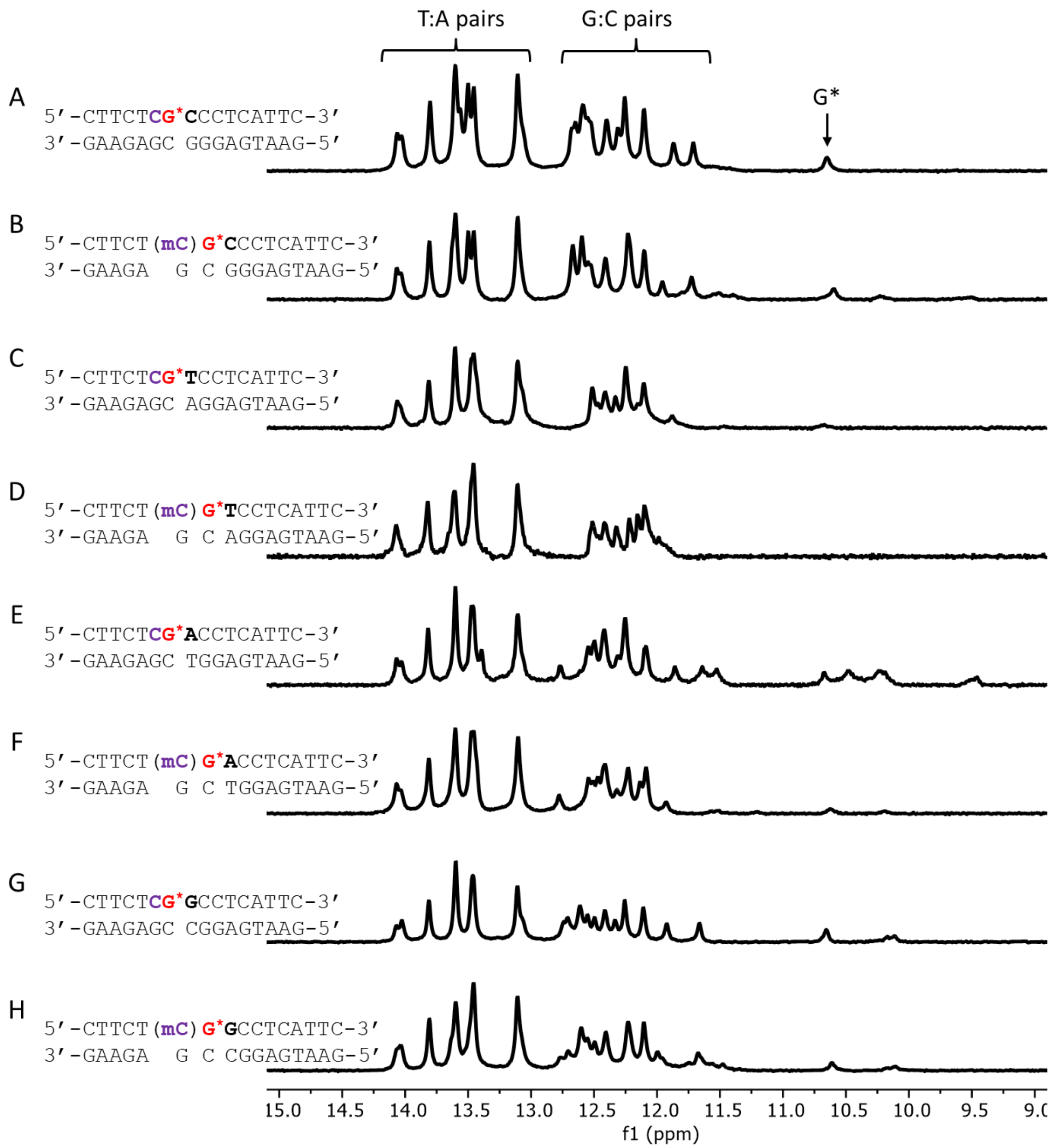


**Figure S11.** Stacked imino proton ($^1$H NMR, 9-15 ppm) spectra taken at 3 °C of the FAF-modified 16mer duplexes in 90% H2O/10% D2O. The thymine imino protons appear between 13-15 ppm and the guanine imino protons appear between 11.5-13 ppm. The FAF-modified guanine appears as an upfield signal around 10.5 ppm. From top to bottom: (A) -CG*C-; (B) -mCG*C-; (C) -CG*T-; (D) -mCG*T-; (E) -CG*A-; (F) -mCG*A-; (G) -CG*G-; (H) -mCG*G-.

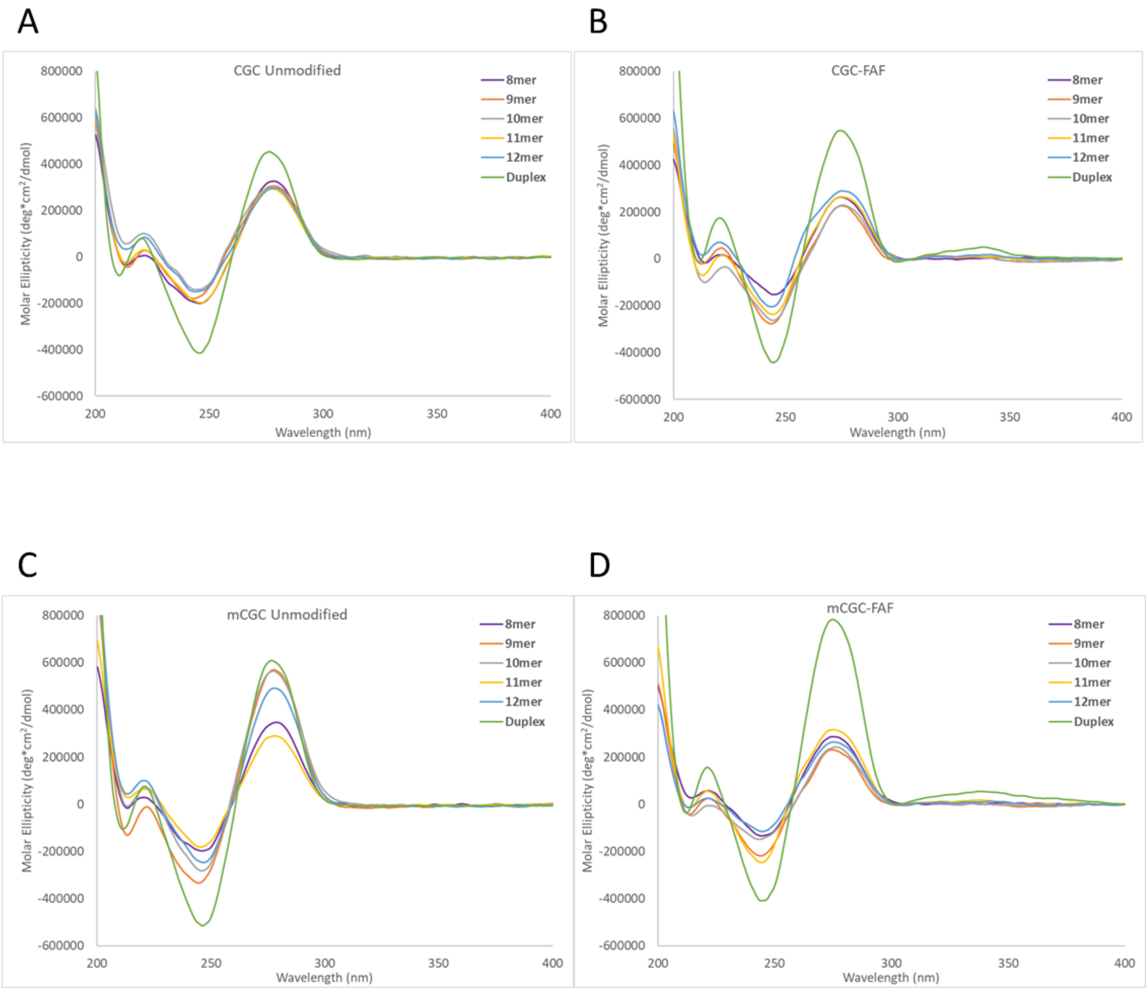


**Figure S12.** Overlaid CD spectra taken at 25°C showing the effect of primer elongation opposite (A) unmodified 16mer CGC; (B) FAF-modified 16mer CGC; (C) unmodified 16mer mCGC; and (D) FAF-modified 16mer mCGC where the lesion position (n) is the 10mer.

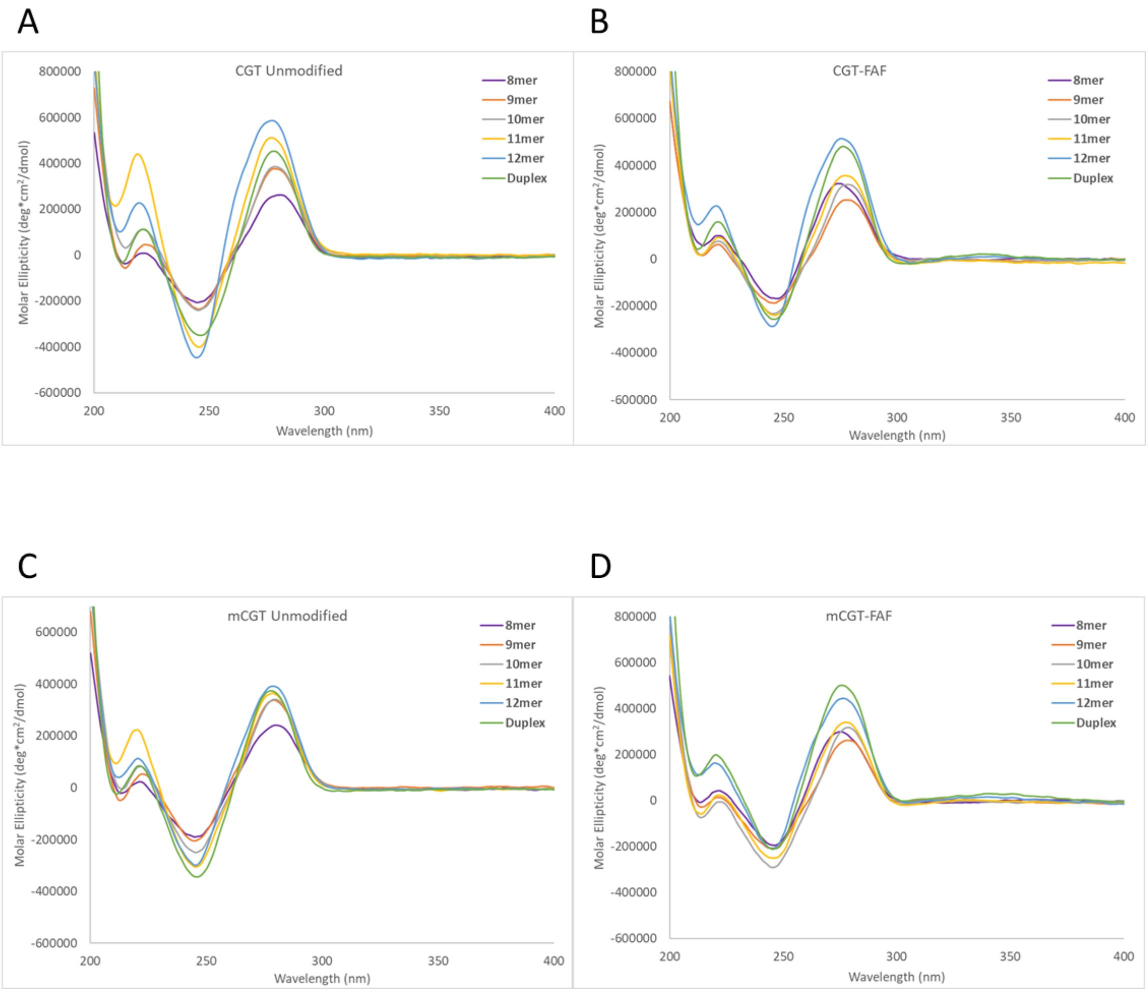


**Figure S13.** Overlaid CD spectra taken at 25°C showing the effect of primer elongation opposite (A) unmodified 16mer CGT; (B) FAF-modified 16mer CGT; (C) unmodified 16mer mCGT; and (D) FAF-modified 16mer mCGT where the lesion position (n) is the 10mer.

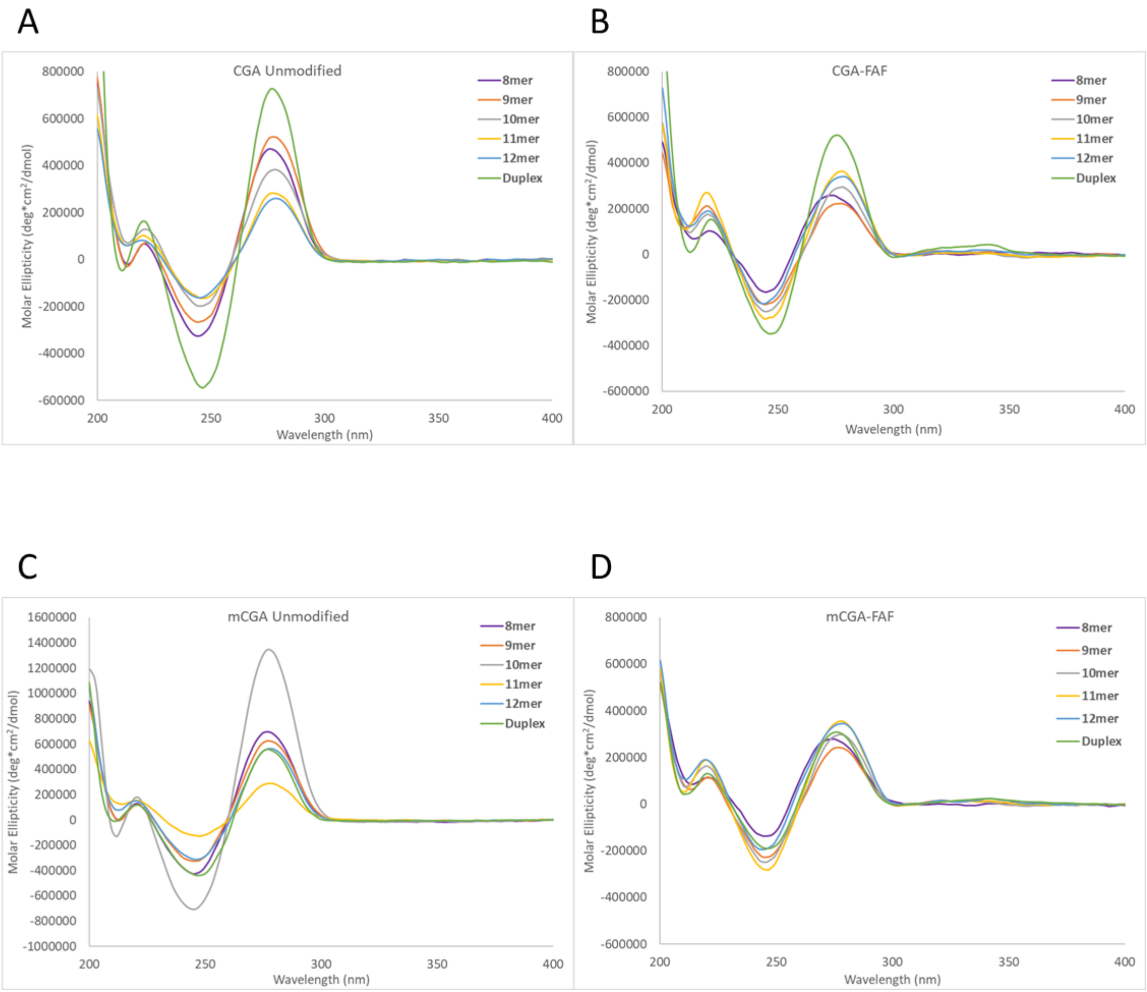


**Figure S14.** Overlaid CD spectra taken at 25°C showing the effect of primer elongation opposite (A) unmodified 16mer CGA; (B) FAF-modified 16mer CGA; (C) unmodified 16mer mCGA; and (D) FAF-modified 16mer mCGA where the lesion position (n) is the 10mer.

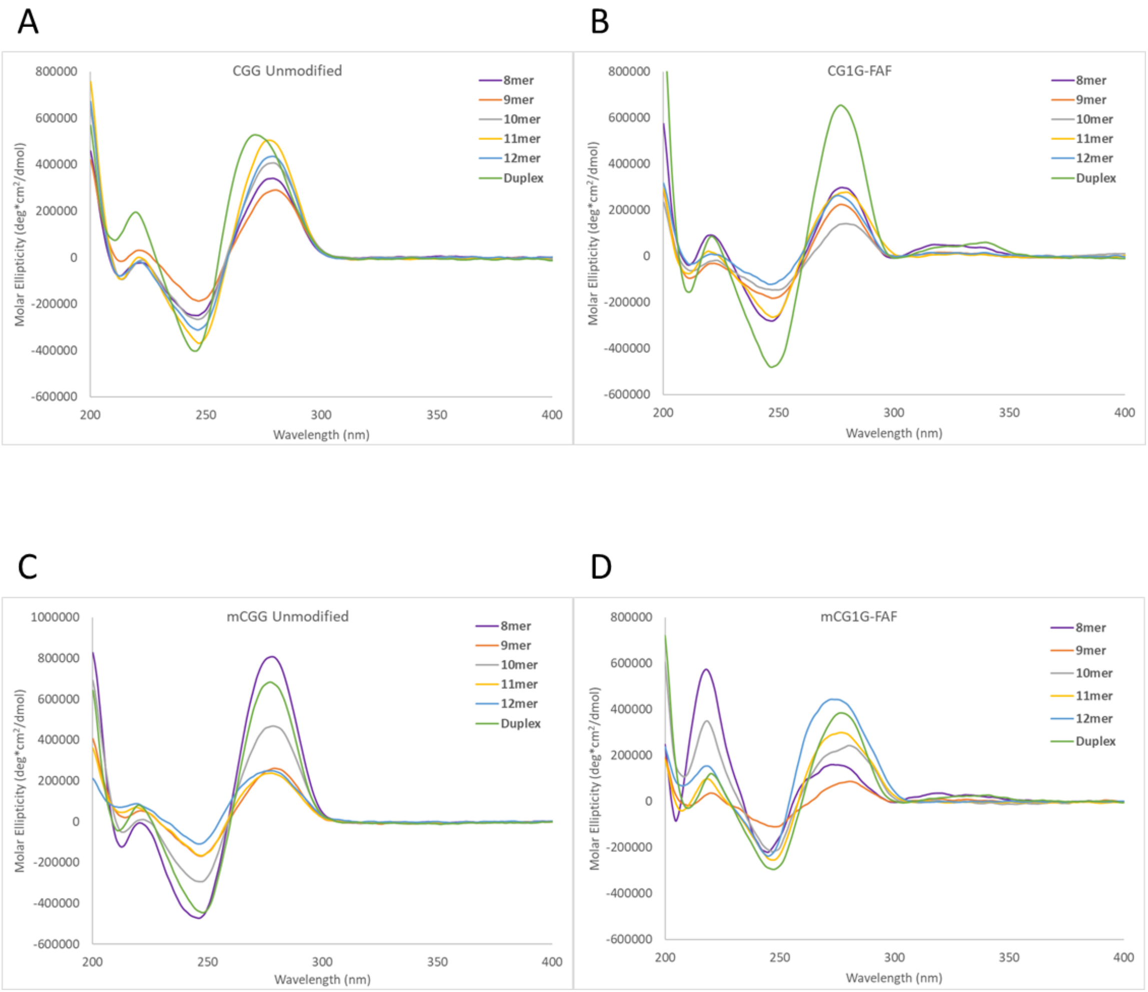


**Figure S15.** Overlaid CD spectra taken at 25°C showing the effect of primer elongation opposite (A) unmodified 16mer CGG; (B) FAF-modified 16mer CGG; (C) unmodified 16mer mCGG; and (D) FAF-modified 16mer mCGG where the lesion position (n) is the 10mer.

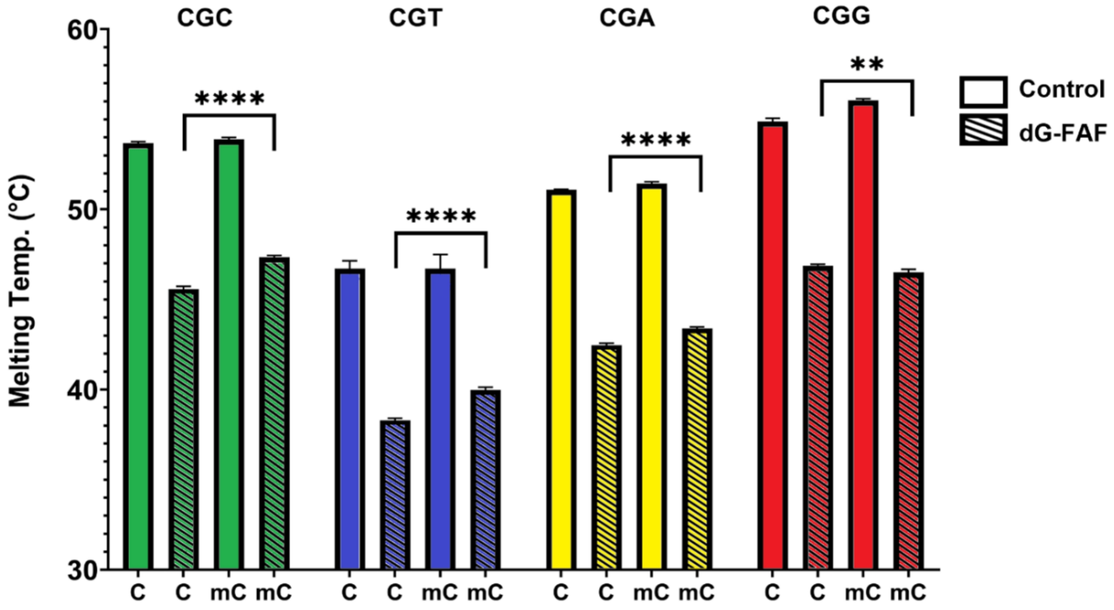


**Figure S16.** Melting temperature (Tm) of the 16mer duplexes. Data presented as the arithmetic mean (n=5) ± standard deviation. X-axis labels indicate whether the 5′ base is C or 5mC and patterns distinguish between unmodified control (solid) versus FAF-modified (G*; slashed) sequences. Statistical significance: ** = P < 0.01, **** = P < 0.00001.

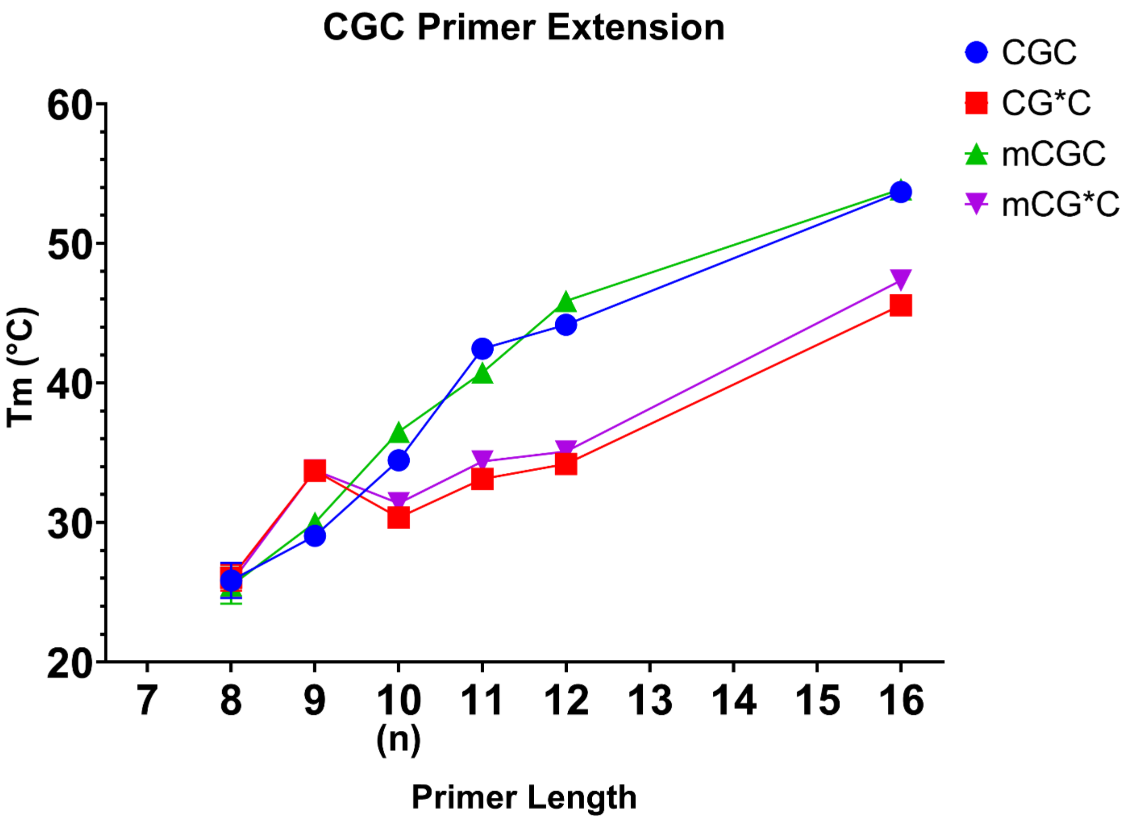


**Figure S17.** UV melting temperature (Tm) of unmodified CGC (blue circles), FAF-modified CG*C (red squares), unmodified mCGC (green triangles), and FAF-modified mCG*C (purple triangles) as a function of primer elongation where the lesion position (n) is the 10mer. Data presented as the arithmetic mean (n=5) ± standard deviation (some error bars may be too small to be visible behind the symbols).

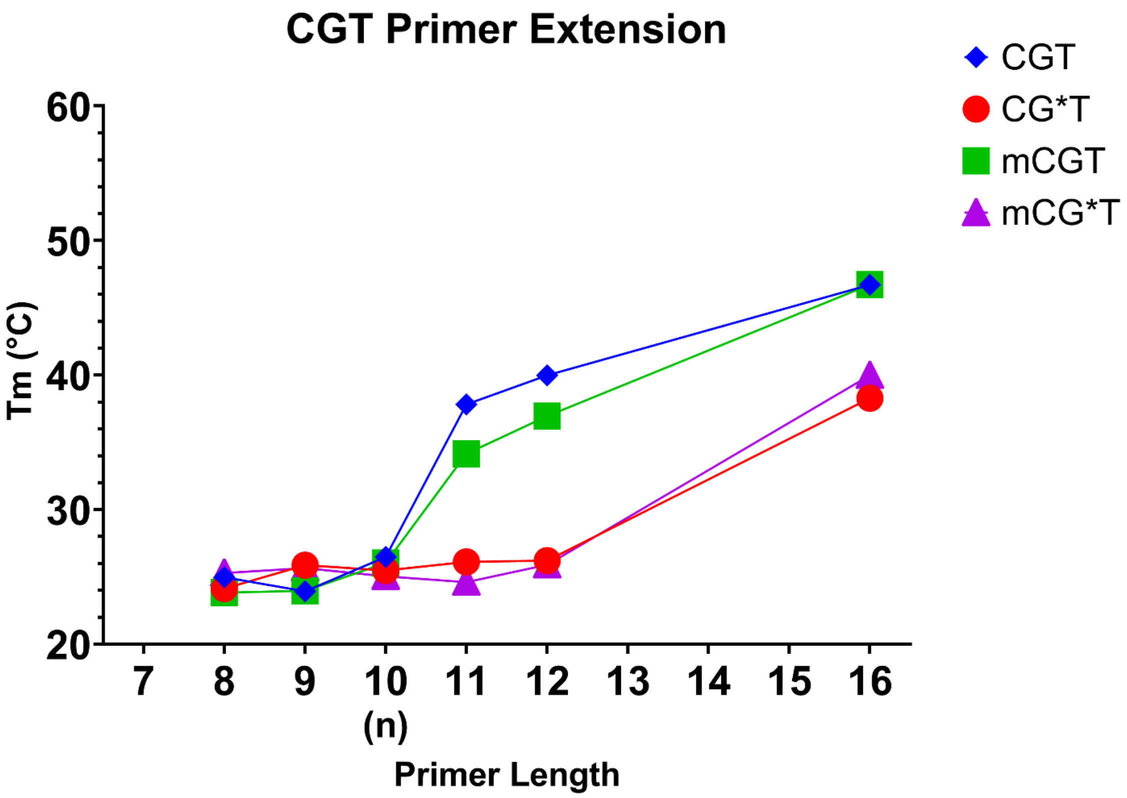


**Figure S18.** UV melting temperature (Tm) of unmodified CGT (blue diamonds), FAF-modified CG*T (red circles), unmodified mCGT (green squares), and FAF-modified mCG*T (purple triangles) as a function of primer elongation where the lesion position (n) is the 10mer. Data presented as the arithmetic mean (n=5) ± standard deviation (some error bars may be too small to be visible behind the symbols).

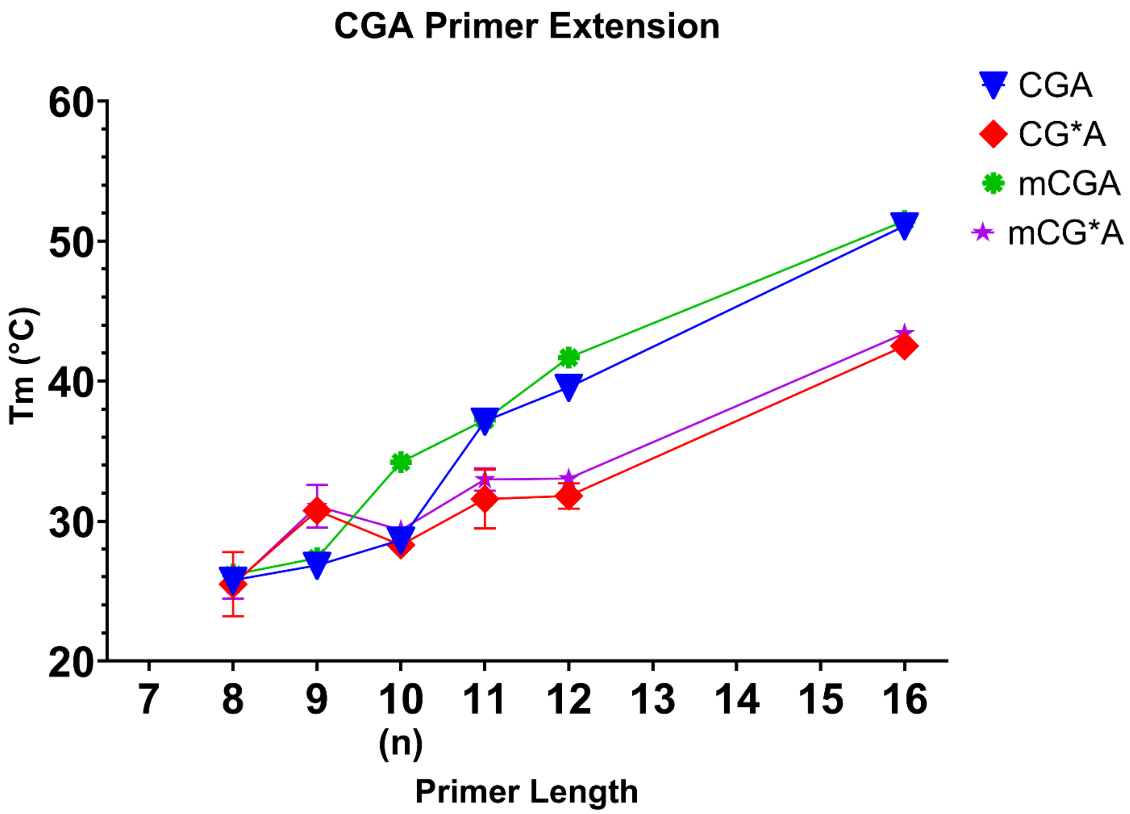


**Figure S19.** UV melting temperature (Tm) of unmodified CGA (blue triangles), FAF-modified CG*A (red diamonds), unmodified mCGA (green asterisks), and FAF-modified mCG*A (purple stars) as a function of primer elongation where the lesion position (n) is the 10mer. Data presented as the arithmetic mean (n=5) ± standard deviation (some error bars may be too small to be visible behind the symbols).

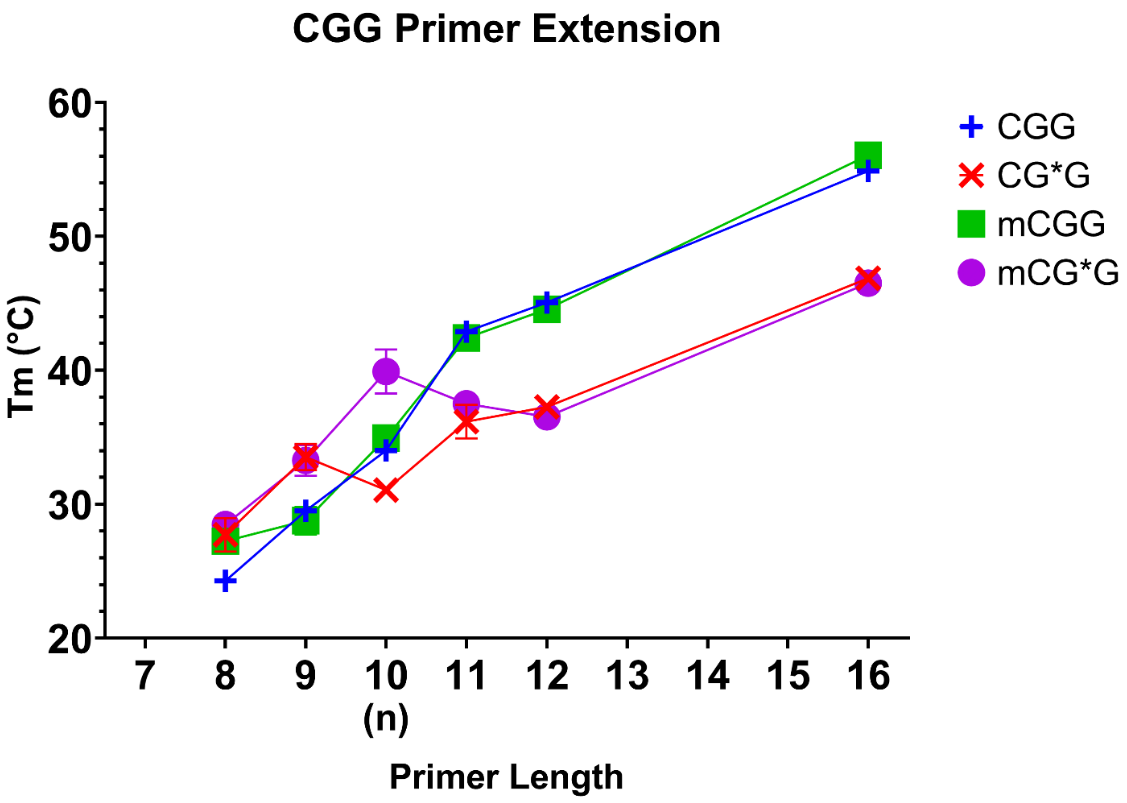


**Figure S20.** UV melting temperature (Tm) of unmodified CGG (blue crosses), FAF-modified CG*G (red x's), unmodified mCGG (green squares), and FAF-modified mCG*G (purple circles) as a function of primer elongation where the lesion position (n) is the 10mer. Data presented as the arithmetic mean (n=5) ± standard deviation (some error bars may be too small to be visible behind the symbols).

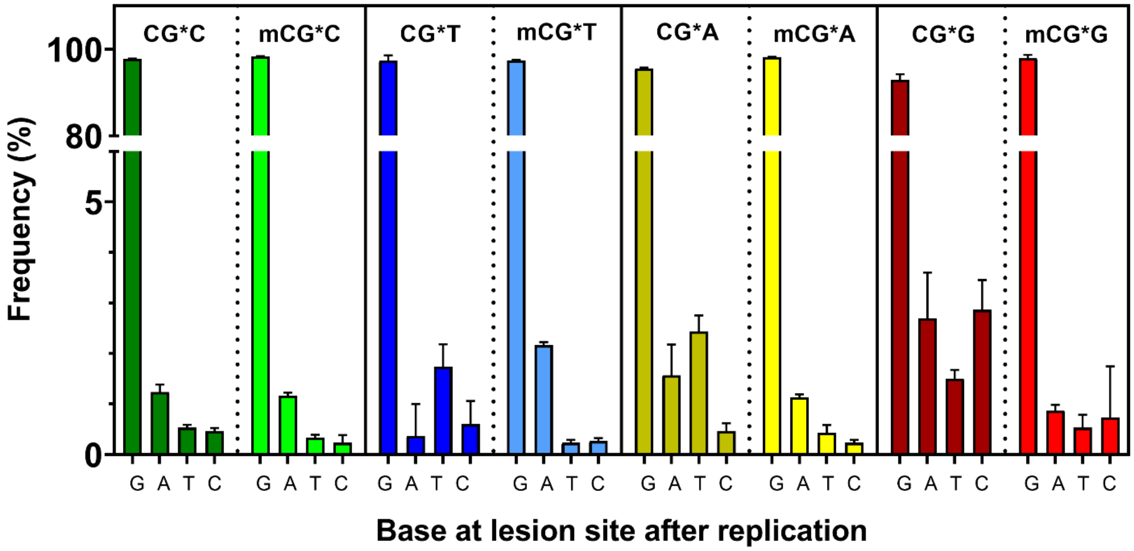


**Figure S21.** Lesion mutational specificity and frequency in HK82 E. coli (AlkB-). Data presented as the arithmetic mean (n=3) ± standard deviation. G* = dG-FAF.

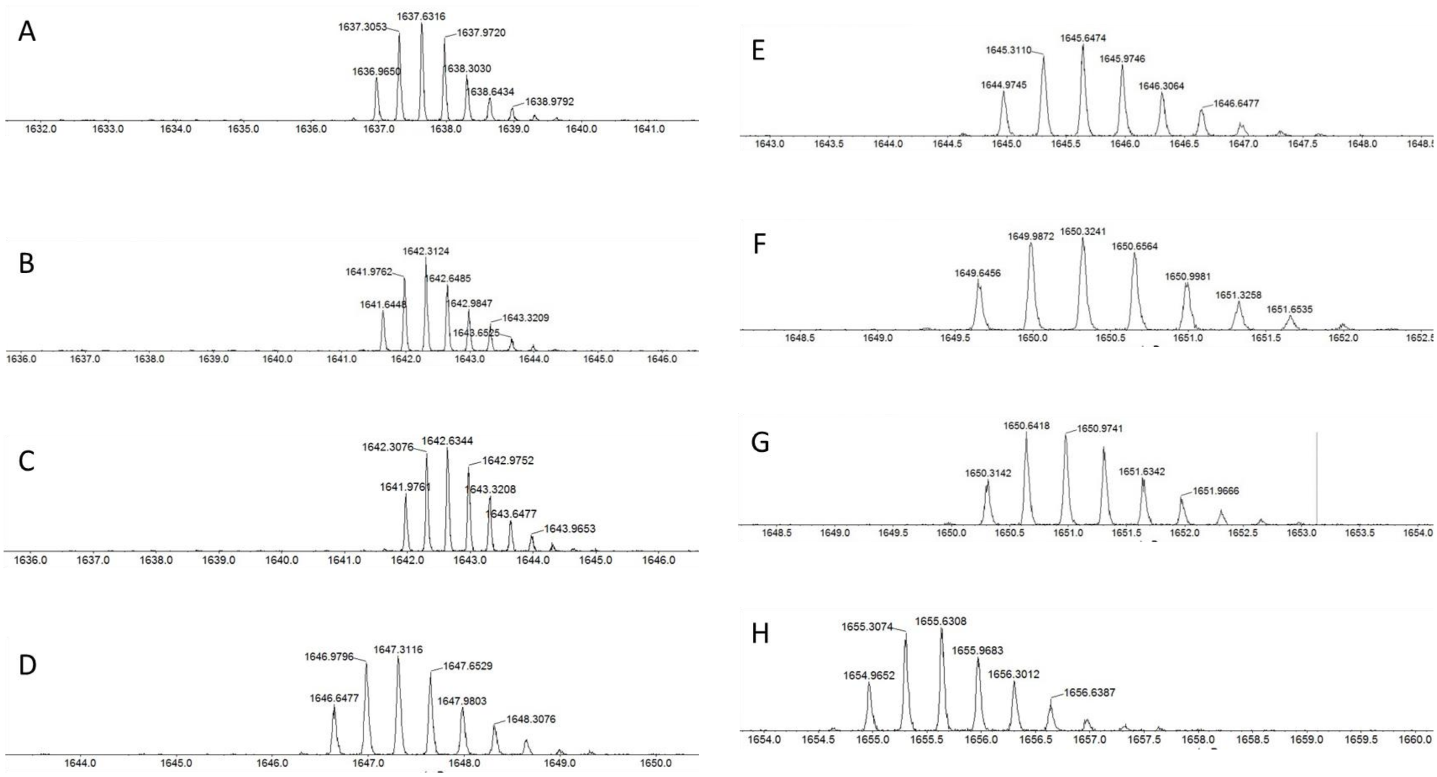


**Figure S22.** ESI-TOF analyses of 16mer oligos containing: (A) CG*C; (B) mCG*C; (C) CG*T; (D) mCG*T; (E) CG*A; (F) mCG*A; (G) CG*G; (H) mCG*G. (G*=dG-C8-FAF). Data represent the −3 charge envelope.

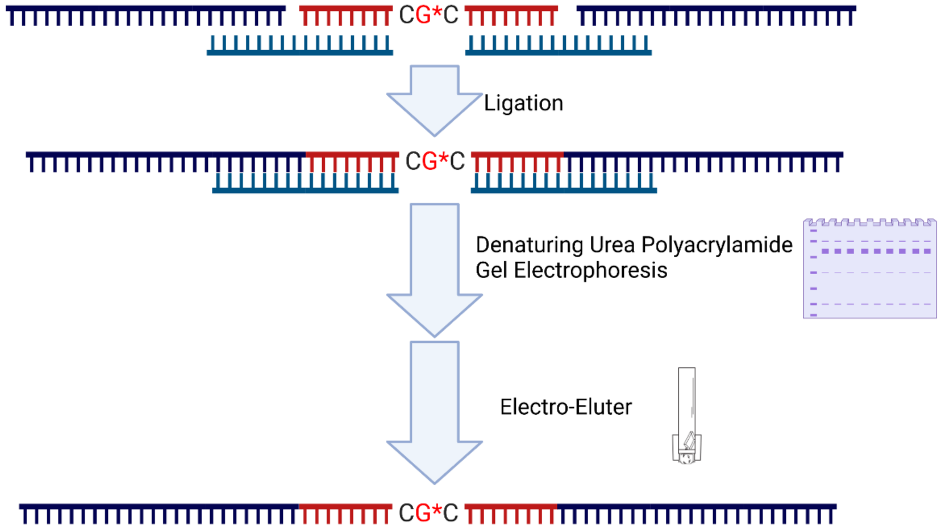


**Figure S23.** Diagram of construction of 58mer lesion-containing oligonucleotide using the CG*C sequence as an illustration (G*=dG-C8-FAF).

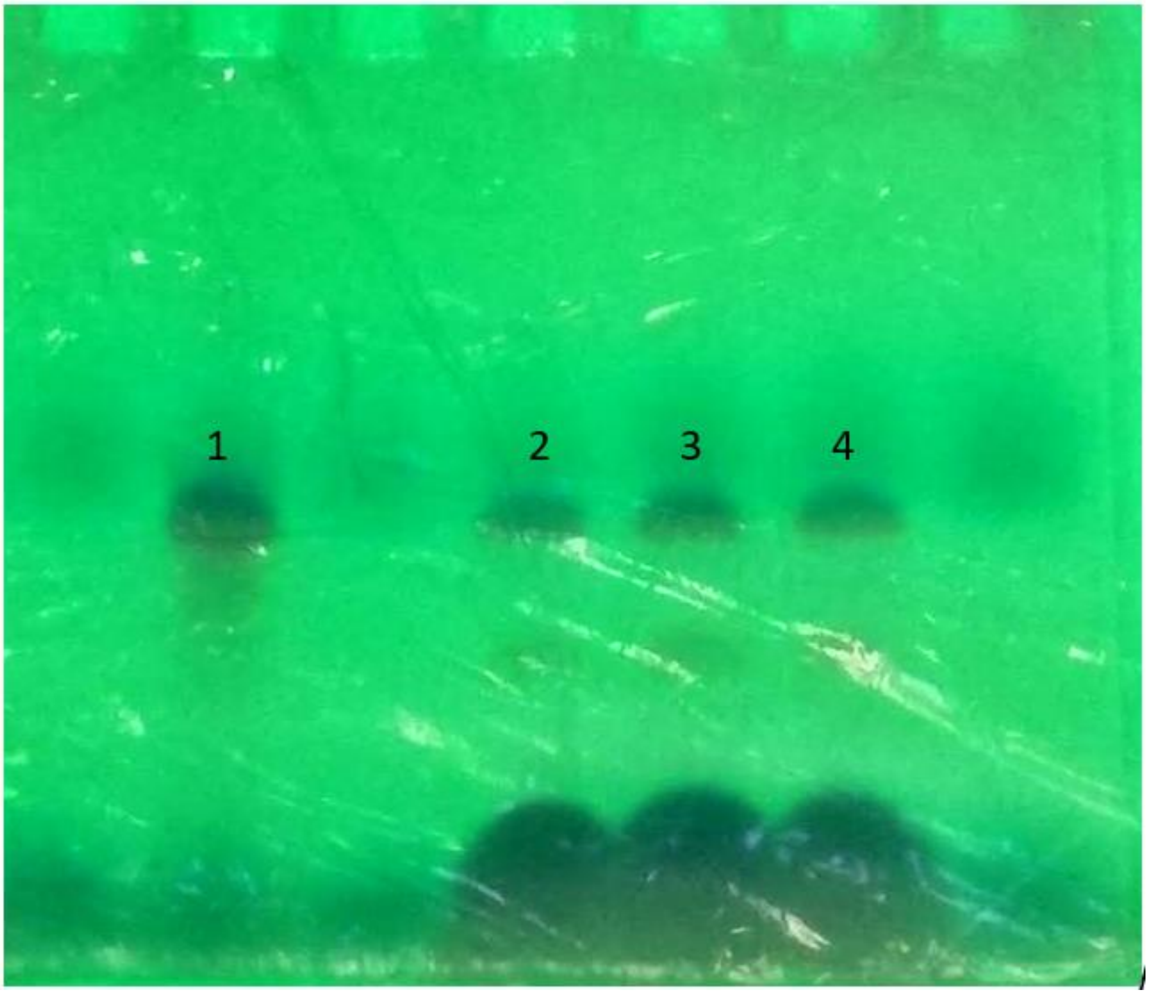


**Figure S24.** Denaturing urea polyacrylamide gel of 58mer lesion-containing oligonucleotide using the CG*C sequence as an illustration (G*=dG-C8-FAF). Lane 1: 58mer control. Lane 2-4: 58mer CG*C ligation product.

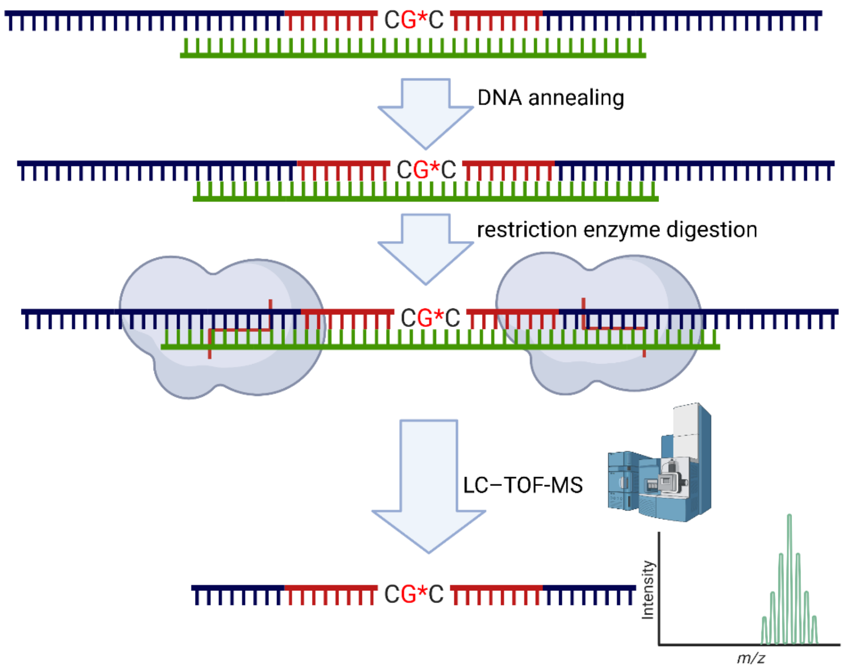


**Figure S25.** Diagram of LC-TOF-MS identification of the digestion product from the 58mer lesion-containing oligonucleotide using the CG*C sequence as an illustration (G*=dG-C8-FAF).

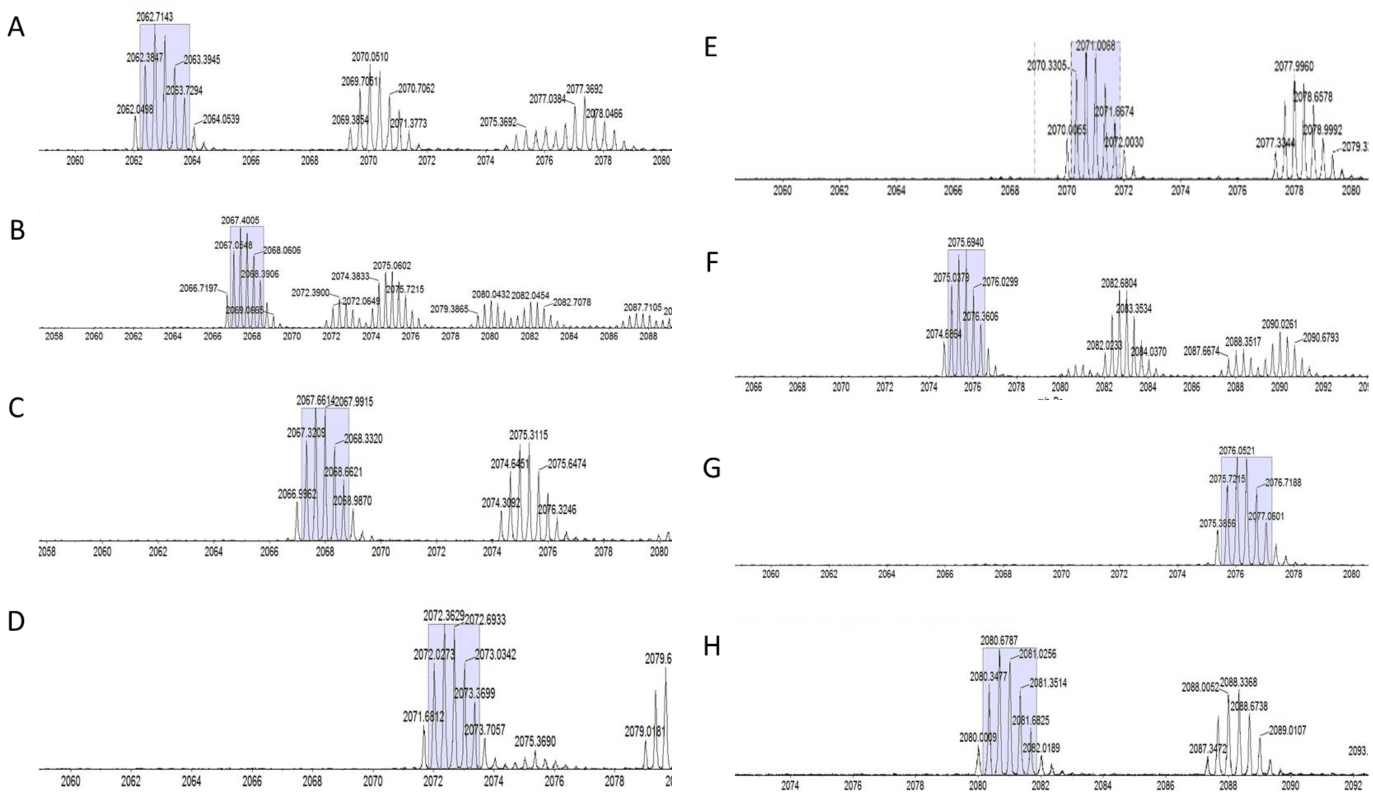


**Figure S26.** ESI-TOF analyses of the digestion products from the 58mer oligos containing (A) CG*C; (B) mCG*C; (C) CG*T; (D) mCG*T; (E) CG*A; (F) mCG*A; (G) CG*G; (H) mCG*G digestion products. G*=dG-C8-FAF. Data represents the −3 charge envelope.

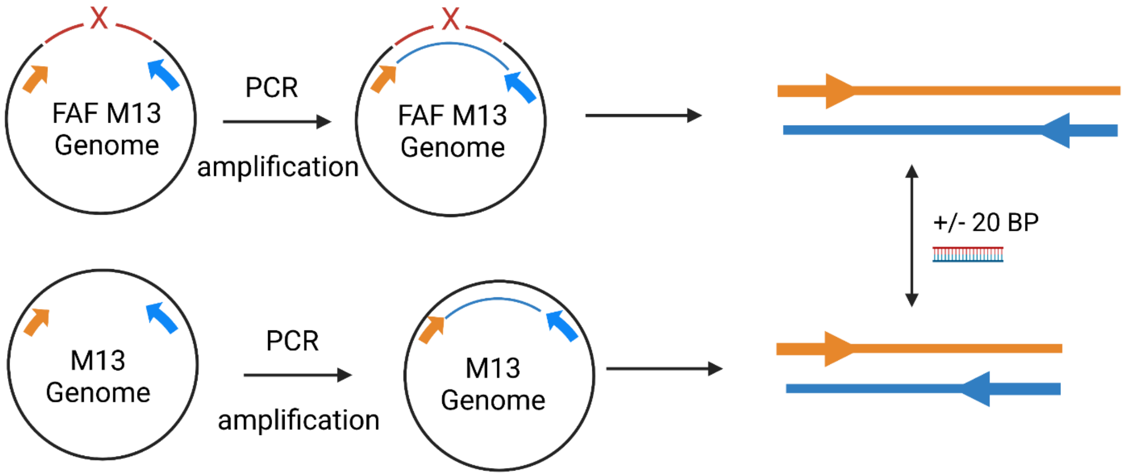


**Figure S27.** Diagram of PCR amplification for lesion containing M13 genome using the CG*C sequence as an illustration (G*=dG-C8-FAF).

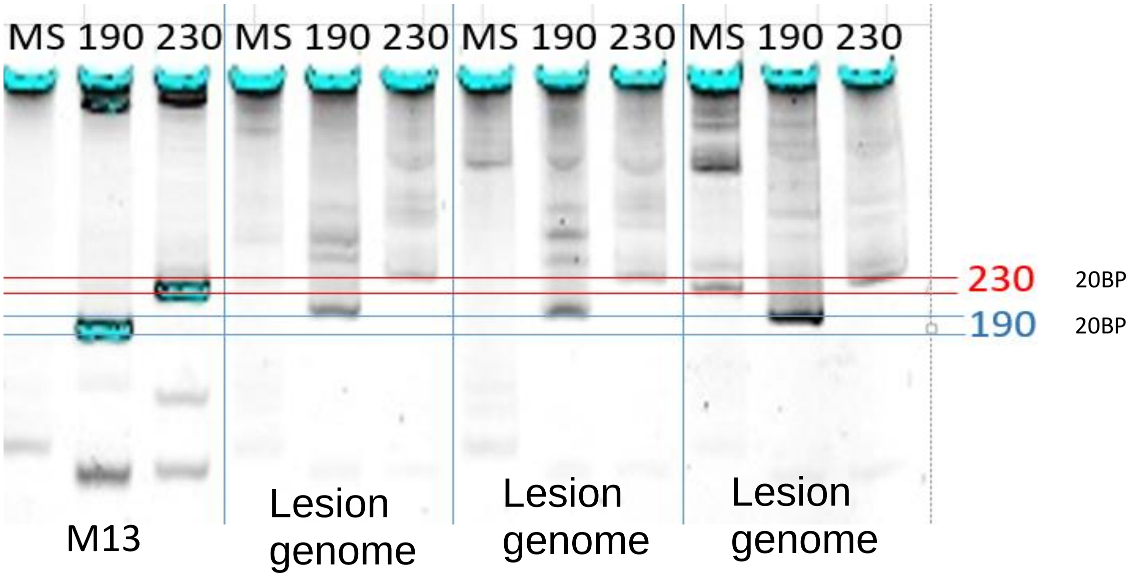


**Figure S28.** Diagram of polyacrylamide gel of PCR products of lesion containing M13 genome using the CG*C sequence with FAF as an illustration. Section 1: 58mer Control. Section 2-4: 58mer CG*C ligation product (G*=dG-C8-FAF). 15% Polyacrylamide gel of FAF-dG containing M13 genome and M13 genome. Two sets of primers were used to generate either 190mer or 230mer DNA product.

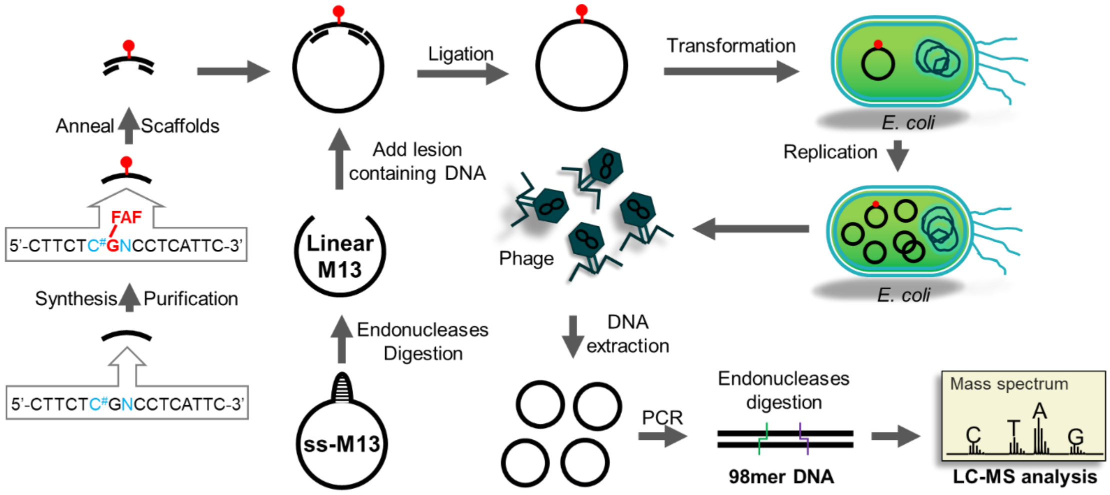


Bypass=$^{\text{Lesion signal/competitor signal}}/_{\text{control signal/competitor signal}}$

Mutation frequency=$^{\text{mutation signal}}/_{\text{all base components signal}}$

**Figure S29.** Diagram of the REAP & CRAB procedures. Sequence C$^{\#}$G*N is used for illustration (G*=dG-C8-FAF).(5)

(5)

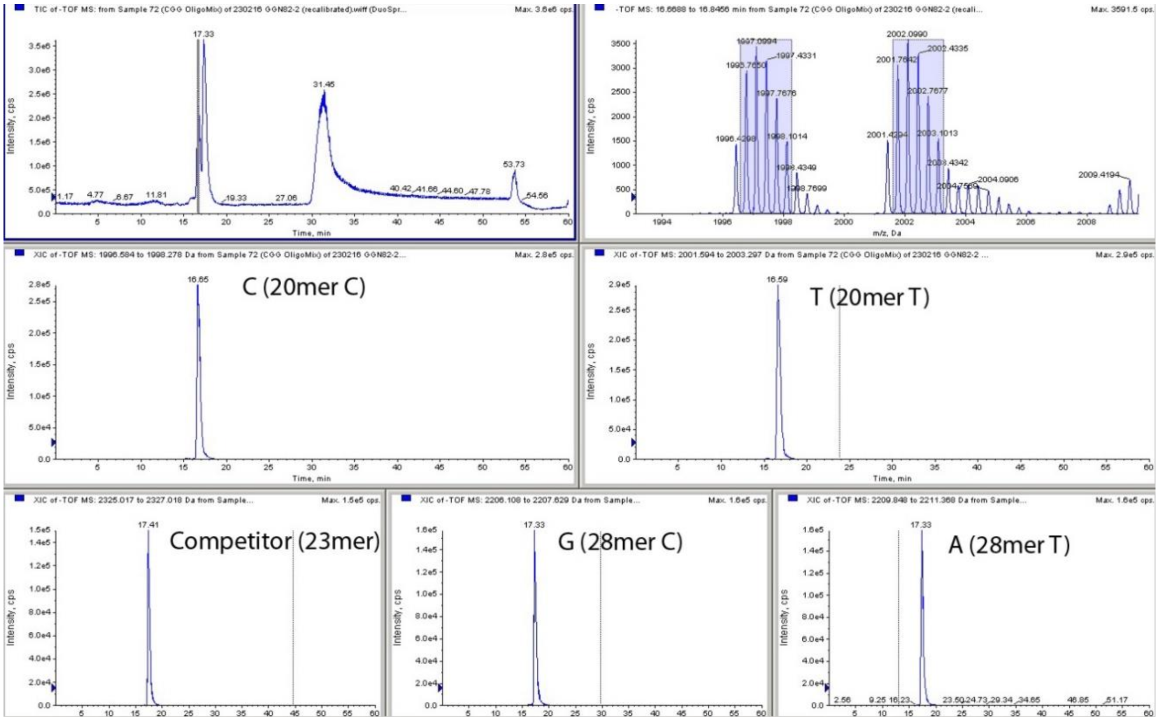


**Figure S30.** A typical LC-TOF-MS spectrum of the REAP and CRAB samples. The C (20mer C), T (20mer T) and Competitor (23mer) are monitored at the -3 charge state. The G (28mer C) and A (28mer T) are monitored at -4 charge state.

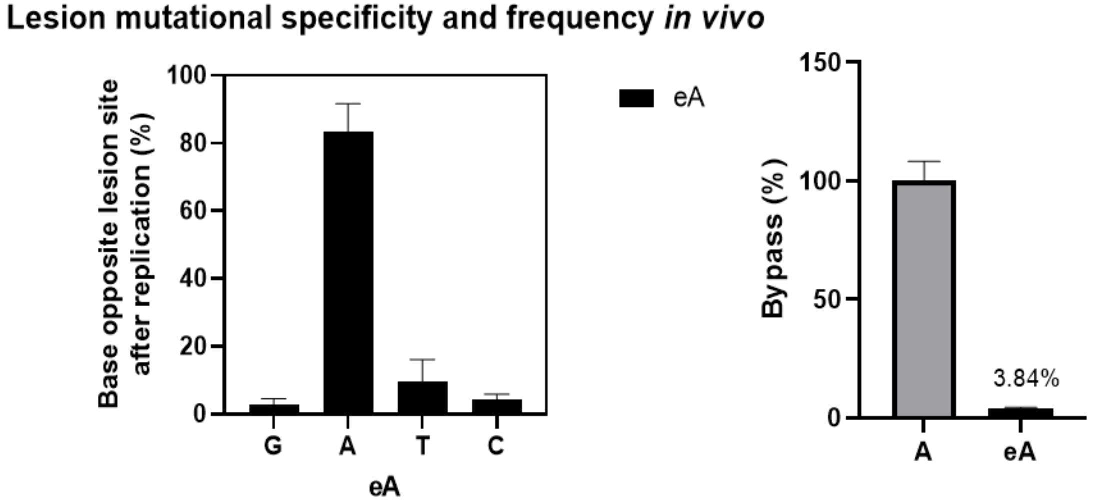


**Figure S31.** A typical LC-TOF-MS analysis of REAP and CRAB samples. eA is used for illustration. Left panel shows mutation patterns of eA and right shows the bypass efficiency of eA.(6)

**Table S1.** Calculated monoisotopic mass and actual m/z measured by MALDI-TOF

| Sequence | Calculated Monoisotopic Mass | | Actual m/z | |
|---|---|---|---|---|
| | Full Length (Da) | Digested (Da) | Full Length | Digested |
| CGT-FAF | 4930.01 | 2244.59 | 4929.90 | 2244.40 |
| mCGT-FAF | 4945.04 | 2259.61 | 4945.10 | 2259.60 |
| CGC-FAF | 4915.01 | 2244.59 | 4919.06 | 2245.92 |
| mCGC-FAF | 4930.04 | 2259.61 | 4934.38 | 2259.69 |
| CGA-FAF | 4939.02 | 2244.59 | 4936.40 | 2244.96 |
| mCGA-FAF | 4954.05 | 2259.61 | 4955.29 | 2258.39 |
| $CG_1G$-FAF | 4955.02 | 2244.59 | 4954.17 | 2245.26 |
| $mCG_1G$-FAF | 4970.04 | 2259.61 | 4968.40 | 2258.38 |
| $CGG_2$-FAF | 4955.02 | 2573.64 | 4954.17 | 2574.51 |
| $mCGG_2$-FAF | 4970.04 | 2588.66 | 4968.40 | 2589.79 |

**Table S2.** Thermal and thermodynamic parameters of FAF-modified duplexes

| ***Template Sequence*** | **Primer Length** | **$\Delta G_{37°C}$ (kcal/mol)[a,b]** | **ΔH (kcal/mol)[a,b]** | **Tm (°C)[a,b]** | **ΔΔG (kcal/mol)[c]** | **ΔΔH (kcal/mol)[d]** | **ΔTm (°C)[e]** |
|---|---|---|---|---|---|---|---|
| CG*C | 8mer | -6.71 | -61.45 | 26.05 | -0.64 | 13.10 | 0.19 |
| | 9mer | -8.30 | -63.89 | 33.72 | -1.07 | 0.07 | 4.67 |
| | 10mer | -7.66 | -58.47 | 30.36 | 0.79 | 9.73 | -4.10 |
| | 11mer | -8.16 | -63.26 | 33.13 | 2.52 | 28.90 | -9.33 |
| | 12mer | -8.36 | -69.98 | 34.19 | 2.64 | 20.79 | -9.98 |
| | 16mer | -11.97 | -113.44 | 45.58 | 3.57 | 16.46 | -8.11 |
| mCG*C | 8mer | -6.78 | -55.82 | 25.70 | -0.55 | 13.21 | 0.22 |
| | 9mer | -8.26 | -62.50 | 33.74 | -0.88 | 4.23 | 3.75 |
| | 10mer | -7.83 | -61.69 | 31.39 | 1.12 | 13.50 | -5.12 |
| | 11mer | -8.38 | -67.41 | 34.39 | 1.76 | 14.72 | -6.37 |
| | 12mer | -8.53 | -75.03 | 35.10 | 3.22 | 25.76 | -10.78 |
| | 16mer | -12.79 | -119.49 | 47.35 | 2.02 | -4.58 | -6.54 |
| CG*T | 8mer | -6.19 | -66.36 | 24.12 | -0.46 | 9.25 | -0.85 |
| | 9mer | -6.16 | -73.98 | 25.86 | -1.38 | 16.49 | 1.94 |
| | 10mer | -6.18 | -70.85 | 25.48 | 0.09 | 3.24 | -1.00 |
| | 11mer | -6.85 | -56.67 | 26.12 | 2.34 | 27.80 | -11.70 |
| | 12mer | -6.75 | -59.63 | 26.20 | 3.26 | 47.80 | -13.77 |
| | 16mer | -8.86 | -86.07 | 38.28 | 4.02 | 43.02 | -8.42 |
| mCG*T | 8mer | -5.94 | -72.58 | 25.29 | -0.25 | 0.54 | 1.46 |
| | 9mer | -6.17 | -71.28 | 25.66 | -1.40 | 20.84 | 1.69 |
| | 10mer | -5.46 | -76.99 | 25.03 | 0.64 | -2.30 | -0.99 |
| | 11mer | -5.42 | -81.87 | 24.60 | 2.76 | 0.88 | -9.55 |
| | 12mer | -5.99 | -77.27 | 25.89 | 2.94 | 18.85 | -11.06 |
| | 16mer | -9.81 | -112.63 | 39.98 | 3.41 | 21.85 | -6.74 |
| CG*A | 8mer | -6.55 | -62.75 | 25.50 | -0.31 | 9.15 | -0.28 |
| | 9mer | -7.59 | -64.97 | 30.75 | -1.17 | 7.94 | 3.91 |
| | 10mer | -7.11 | -62.86 | 28.29 | -0.16 | 6.51 | -0.36 |
| | 11mer | -7.77 | -70.13 | 31.57 | 1.33 | 14.62 | -5.57 |
| | 12mer | -7.58 | -79.62 | 31.80 | 2.17 | 15.14 | -7.77 |
| | 16mer | -10.87 | -111.48 | 42.51 | 3.21 | 7.55 | -8.58 |
| mCG*A | 8mer | -6.44 | -68.61 | 25.41 | 0.19 | -4.85 | -0.75 |
| | 9mer | -7.38 | -76.81 | 31.06 | -0.68 | -8.11 | 3.70 |
| | 10mer | -7.30 | -65.78 | 29.36 | 1.08 | 12.36 | -4.85 |
| | 11mer | -8.04 | -71.46 | 32.97 | 1.06 | 15.04 | -4.26 |
| | 12mer | -8.00 | -74.25 | 33.04 | 2.31 | 18.75 | -8.65 |
| | 16mer | -11.06 | -106.18 | 43.40 | 3.35 | 17.52 | -8.04 |
| CG*G | 8mer | -6.65 | -77.58 | 27.72 | -0.82 | -2.43 | 3.44 |
| | 9mer | -8.21 | -68.65 | 33.53 | -0.93 | -2.01 | 4.02 |
| | 10mer | -7.74 | -64.47 | 31.06 | 0.58 | 6.28 | -2.96 |
| | 11mer | -8.71 | -75.11 | 36.16 | 2.01 | 14.22 | -6.72 |
| | 12mer | -9.09 | -68.05 | 37.27 | 2.32 | 27.43 | -7.80 |
| | 16mer | -12.45 | -114.39 | 46.87 | 6.20 | 61.72 | -8.01 |
| mCG*G | 8mer | -7.35 | -57.57 | 28.49 | -0.72 | 13.47 | 1.24 |
| | 9mer | -8.21 | -62.78 | 33.27 | -1.03 | 2.11 | 4.50 |

| | | | | | | |
|---|---|---|---|---|---|---|
| 10mer | -9.64 | -68.88 | 39.90 | -1.07 | 1.67 | 4.96 |
| 11mer | -8.94 | -46.58 | 37.50 | 2.06 | 60.89 | -4.91 |
| 12mer | -8.88 | -61.54 | 36.53 | 1.95 | 15.97 | -8.03 |
| 16mer | -12.24 | -112.11 | 46.51 | 4.86 | 29.31 | -9.54 |

[a]*Average standard deviations for ΔG, ΔH, and Tm are ±0.16, ±4.25, ±0.44 respectively (n=5).*

[b]*Values calculated from the thermal melt curves.*

[c]$\Delta\Delta G = \Delta G_{modified} - \Delta G_{unmodified}$

[d]$\Delta\Delta H = \Delta H_{modified} - \Delta H_{unmodified}$

[e]$\Delta Tm = Tm_{modified} - Tm_{unmodified}$

**Table S3.** List of oligonucleotide and primer sequences (5′→3′) used for the REAP and CRAB assays

| | | |
|---|---|---|
| CGC | CGC Control | CTTCTCGCCCTCATTC |
| | 3′end oligo | TACCGTCGAGACGCGCATGCA |
| | 5′end oligo | TCTCGAGTGCATCGTCAGCAC |
| | CG*C | CTTCTCG*CCCTCATTC |
| | 3′end oligo | TACCGTCGGTTCGCGCATGCA |
| | 5′end oligo | TCTCGAGTGGTTCGTCAGCAC |
| | mCG*C | CTTCTmCG*CCCTCATTC |
| | 3′end oligo | TACCGTCGCATCGCGCATGCA |
| | 5′end oligo | TCTCGAGTGGTTCGTCAGCAC |
| | Complementary | CACTCGAGAGAATGAGGGCGAGAAGTGCATGCGCG |
| CGT | Control | CTTCTCGTCCTCATTC |
| | 3′end oligo | TACCGTCGAACCGCGCATGCA |
| | 5′end oligo | TCTCGAGTGACTCGTCAGCAC |
| | CG*T | CTTCTCG*TCCTCATTC |
| | 3′end oligo | TACCGTCGGGCCGCGCATGCA |
| | 5′end oligo | TCTCGAGTGGTTCGTCAGCAC |
| | mCG*T | CTTCTmCG*TCCTCATTC |
| | 3′end oligo | TACCGTCGCGGCGCGCATGCA |
| | 5′end oligo | TCTCGAGTGGTTCGTCAGCAC |
| | Complementary | CACTCGAGAGAATGAGGACGAGAAGTGCATGCGCG |
| CGA | Control | CTTCTCGACCTCATTC |
| | 3′end oligo | TACCGTCGGTTCGCGCATGCA |
| | 5′end oligo | TCTCGAGTGCATCGTCAGCAC |
| | CG*A | CTTCTCG*ACCTCATTC |
| | 3′end oligo | TACCGTCGATGCGCGCATGCA |
| | 5′end oligo | TCTCGAGTGGTTCGTCAGCAC |
| | mCG*A | CTTCTmCG*ACCTCATTC |
| | 3′end oligo | TACCGTCGAGACGCGCATGCA |
| | 5′end oligo | TCTCGAGTGGTTCGTCAGCAC |
| | Complementary | GCGCGTACGTGAAGAGCTGGAGTAAGAGAGCTCAC |
| CGG | Control | CTTCTCGGCCTCATTC |
| | 3′end oligo | TACCGTCGTCACGCGCATGCA |
| | 5′end oligo | TCTCGAGTGACTCGTCAGCAC |
| | CG*G | CTTCTCG*GCCTCATTC |
| | 3′end oligo | TACCGTCGGAACGCGCATGCA |
| | 5′end oligo | TCTCGAGTGGTTCGTCAGCAC |
| | mCG*G | CTTCTCG*GCCTCATTC |
| | 3′end oligo | TACCGTCGACTCGCGCATGCA |
| | 5′end oligo | TCTCGAGTGGTTCGTCAGCAC |

| | | |
|---|---|---|
| | Complementary | GCGCGTACGTGAAGAGCCGGAGTAAGAGAGCTCAC |
| FAF Competitor | 19mer | CTTCTTGACCTCATTCTAG |
| | 3'end oligo | TACCGTCGCTACGCGCATGCA |
| | 5'end oligo | TCTCGAGTGTGTCGTCAGCAC |
| eA | eA Control | GAAGACCTAGGCGTCC |
| | 3'end oligo | TACCGTCGTTCCGCGCATGCA |
| | 5'end oligo | TCTCGAGTGAACCGTCAGCAC |
| | eA | GAAGACCTA*GGCGTCC |
| | 3'end oligo | TACCGTCGTAGCGCGCATGCA |
| | 5'end oligo | TCTCGAGTGAACCGTCAGCAC |
| | eACompetitor | GAAGACCTAGGCGTCCTAG |
| | 3'end oligo | TACCGTCGATGCGCGCATGCA |
| | 5'end oligo | TCTCGAGTGAACCGTCAGCAC |
| Scaffold I | | AGAAGTGCATGCGCG |
| Scaffold II | | CACTCGAGAGAATGAGG |
| 190 primer Forward | | TTGTGTGGAATTGTGAGCGG |
| 190 primer Reverse | | TGCAAGGCGATTAAGTTGGG |
| 230 primer Forward | | CACCCCAGGCTTTACACTTT |
| 230 primer Reverse | | GCAAGGCGATTAAGTTGGGTAA |
| MS primer | | CGCCAGGGTTTTCCCAGTCACGAC |
| MS primer | | AGCGGATAACAATTTCACACAGGA |

**Table S4.** Calculated and observed monoisotopic MW and m/z value of modified oligonucleotides. (G*=dG-C8-FAF)

| 5'-CTTCT**$\underline{C^{\#}G^{*}N}$**CCTCATTC-3' | MW (calculated) of neutral species | m/z (calculated) -3 charge peak | m/z (observed) -3 charge peak | Mass accuracy (ppm) |
|---|---|---|---|---|
| 16mer CG*C | 4913.8657 | 1636.9474 | 1636.9650 | 10.75 |
| 16mer CG*T | 4928.8654 | 1641.9473 | 1641.9761 | 17.54 |
| 16mer CG*A | 4937.8769 | 1644.9511 | 1644.9745 | 14.20 |
| 16mer CG*G | 4953.8718 | 1650.2828 | 1650.3142 | 19.04 |
| 16mer mCG*C | 4927.8813 | 1641.6193 | 1641.6448 | 15.54 |
| 16mer mCG*T | 4942.8810 | 1646.6192 | 1646.6477 | 17.32 |
| 16mer mCG*A | 4951.8926 | 1649.6230 | 1649.6456 | 13.68 |
| 16mer mCG*G | 4967.8875 | 1654.9547 | 1654.9652 | 6.36 |

**Table S5.** Calculated and observed monoisotopic MW and m/z value of modified oligonucleotides after digestion. (G*=dG-C8-FAF) (5, 6)

| | MW (calculated) of neutral species | m/z (calculated) -3 charge peak | m/z (observed) -3 charge peak | Mass accuracy (ppm) |
|---|---|---|---|---|
| 20mer CG*C | 6189.0284 | 2062.0016 | 2062.0498 | 23.35 |
| 20mer CG*T | 6204.0281 | 2067.0015 | 2066.9962 | -2.58 |
| 20mer CG*A | 6213.0397 | 2070.0054 | 2070.0055 | 0.05 |
| 20mer CG*G | 6229.0346 | 2075.3370 | 2075.3856 | 23.40 |
| 20mer mCG*C | 6203.0441 | 2066.6735 | 2066.7197 | 22.34 |
| 20mer mCG*T | 6218.0437 | 2071.6734 | 2071.6812 | 3.76 |
| 20mer mCG*A | 6227.0553 | 2074.6773 | 2074.6864 | 4.40 |
| 20mer mCG*G | 6243.0502 | 2080.0089 | 2080.0009 | -3.85 |

## Supplemental References

1. Marques,M.M. and Beland,F.A. (1990) Synthesis, characterization, and solution properties of ras sequences modified by arylamine carcinogens at the first base of codon 61. *Chem. Res. Toxicol.*, **3**, 559–565.
2. Miller,E.C., Juhl,U. and Miller,J.A. (1966) Nucleic Acid Guanine: Reaction with the Carcinogen N-Acetoxy-2-Acetylaminofluorene. *Science (1979)*, **153**, 1125–1127.
3. Zhou,L., Rajabzadeh,M., Traficante,D.D. and Cho,B.P. (1997) Conformational Heterogeneity of Arylamine-Modified DNA: 19 F NMR Evidence. *J. Am. Chem. Soc.*, **119**, 5384–5389.
4. Shrivastav,N., Fedeles,B.I., Li,D., Delaney,J.C., Frick,L.E., Foti,J.J., Walker,G.C. and Essigmann,J.M. (2014) A chemical genetics analysis of the roles of bypass polymerase DinB and DNA repair protein AlkB in processing N2-alkylguanine lesions in vivo. *PLoS One*, **9**.
5. Delaney,J.C. and Essigmann,J.M. (2006) Assays for Determining Lesion Bypass Efficiency and Mutagenicity of Site-Specific DNA Lesions In Vivo. *Meth. Enzymol.*, **408**, 1–15.
6. Delaney,J.C., Smeester,L., Wong,C., Frick,L.E., Taghizadeh,K., Wishnok,J.S., Drennan,C.L., Samson,L.D. and Essigmann,J.M. (2005) AlkB reverses etheno DNA lesions caused by lipid oxidation in vitro and in vivo. *Nat. Struct. Mol. Biol.*, **12**, 855–860.
